# Soil Calcium Availability Influences Shell Ecophenotype Formation in the Sub-Antarctic Land Snail, *Notodiscus hookeri*

**DOI:** 10.1371/journal.pone.0084527

**Published:** 2013-12-20

**Authors:** Maryvonne Charrier, Arul Marie, Damien Guillaume, Laurent Bédouet, Joseph Le Lannic, Claire Roiland, Sophie Berland, Jean-Sébastien Pierre, Marie Le Floch, Yves Frenot, Marc Lebouvier

**Affiliations:** 1 Université de Rennes 1, Université Européenne de Bretagne, UMR CNRS 6553, Campus de Beaulieu, Rennes, France; 2 Muséum National d’Histoire Naturelle, Plateforme de Spectrométrie de Masse et de Protéomique, UMR CNRS 7245, Département Régulation Développement et Diversité Moléculaire, Paris, France; 3 Université de Toulouse, Observatoire Midi-Pyrénées, Géosciences Environnement Toulouse, UMR 5563 (CNRS/UPS/IRD/CNES), Toulouse, France.; 4 Muséum National d’Histoire Naturelle, Biologie des Organismes et Ecosystèmes Aquatiques, UMR CNRS 7208 / IRD 207, Paris, France; 5 Université de Rennes 1, Université Européenne de Bretagne, Service Commun de Microscopie Electronique à Balayage et micro-Analyse, Rennes, France; 6 Université de Rennes 1, Université Européenne de Bretagne, Sciences Chimiques de Rennes, UMR CNRS 6226, Campus de Beaulieu, Rennes, France; 7 Institut Polaire Français Paul Émile Victor, Technopôle Brest-Iroise, Plouzané, France; 8 Université de Rennes 1, Université Européenne de Bretagne, UMR CNRS 6553, Station Biologique, Paimpont, France; Faculdade de Medicina Dentária, Universidade do Porto, Portugal

## Abstract

Ecophenotypes reflect local matches between organisms and their environment, and show plasticity across generations in response to current living conditions. Plastic responses in shell morphology and shell growth have been widely studied in gastropods and are often related to environmental calcium availability, which influences shell biomineralisation. To date, all of these studies have overlooked micro-scale structure of the shell, in addition to how it is related to species responses in the context of environmental pressure. This study is the first to demonstrate that environmental factors induce a bi-modal variation in the shell micro-scale structure of a land gastropod. *Notodiscus hookeri* is the only native land snail present in the Crozet Archipelago (sub-Antarctic region). The adults have evolved into two ecophenotypes, which are referred to here as MS (mineral shell) and OS (organic shell). The MS-ecophenotype is characterised by a thick mineralised shell. It is primarily distributed along the coastline, and could be associated to the presence of exchangeable calcium in the clay minerals of the soils. The Os-ecophenotype is characterised by a thin organic shell. It is primarily distributed at high altitudes in the mesic and xeric fell-fields in soils with large particles that lack clay and exchangeable calcium. Snails of the Os-ecophenotype are characterised by thinner and larger shell sizes compared to snails of the MS- ecophenotype, indicating a trade-off between mineral thickness and shell size. This pattern increased along a temporal scale; whereby, older adult snails were more clearly separated into two clusters compared to the younger adult snails. The prevalence of glycine-rich proteins in the organic shell layer of *N. hookeri*, along with the absence of chitin, differs to the organic scaffolds of molluscan biominerals. The present study provides new insights for testing the adaptive value of phenotypic plasticity in response to spatial and temporal environmental variations.

## Introduction

Changes in morphology, behaviour and physiology are all related to the ecological constraints placed on organisms [1,2]. In heterogeneous environments, a large range of adaptive traits is likely to benefit organisms that use a broad range of habitats [2]. Alternatively, organisms that have a sedentary life interrupted by cyclic movements may adopt a flexible phenotype [3]. In addition, focal environmental variation may favour adaptation to local conditions for nested populations [4]. Consequently a species that is widespread in heterogeneous environments, with populations that are restricted to focal habitats due to their poor dispersal ability, are expected to evolve into either (i) locally adapted ecophenotypes, if the phenotypes are induced by the environment and show plasticity across generations in response to their currently living conditions or, (ii) ecotypes, if variation is supported by a genetic basis (i.e. heritable variation in traits) [4]. 

In gastropods, ecophenotypes and ecotypes have received wide attention due to the extraordinary diversity of shell morphology within the species. For example, changes in shell shape have been related to altitudinal and latitudinal gradients [5,6], shore environments and predator-prey interactions [4,7], water and habitat characteristics [8] and hybridisation [9]. Shell regression has also been well studied, particularly among cephalopods and gastropods, which have internalised shells [10]. In opisthobranch gastropods, the poor calcification of shells is counterbalanced by high levels of chitin [11]. Shell reduction or loss is a trait that is usually correlated with a shift from mechanical shell protection to chemical body defence [12]. Based on evidence demonstrating that many gastropods exhibit outer shell plasticity, we predict that the micro-scale structure of the shell might also respond to sharp habitat changes. In fact, the molluscan shell has a complex organic matrix that provides a framework for sustained and highly organised calcium carbonate crystallisation [13]. The mineralisation process may be metabolically expensive, particularly at cold temperatures, resulting in decreased skeletal mass, as reported for marine invertebrates [14]. In addition, a reduction in shell thickness is not necessarily related to an increase in latitude, but is, rather, influenced by ecological factors, as documented for brachiopods and laternulid clams [14]. In the case of marine molluscs, higher CaCO_3_ dissolution in the surface waters of temperate oceans might act as source of calcium, diminishing the burden of biomineralisation [14]. However, under terrestrial conditions, access to free calcium ions is generally limited and, consequently, mineral resources in the soil and food are thought to largely influence shell biomineralisation.

Due to severe environmental conditions, the sub-Antarctic islands have low terrestrial biodiversity, resulting in their containing relatively simple ecosystems [15,16]. Possession Island, which is in the Crozet Archipelago, has a mountainous topography across very short distances (18 km long, 15 km wide), resulting in strong climatic gradients and heterogeneous environments [17]. Among the native invertebrates that are represented by about 50 species, only one terrestrial snail, *Notodiscus hookeri* [18], has been recorded, and is widespread on this island. Large intraspecific variations in shell morphometrics have been reported for this species on Possession Island [6], with endemic variants being described as local adaptations to environmentally distinct islands [19]. In addition, plastic responses in shell morphology and shell growth have been related to calcium availability, a constraint that has implications for molluscan ecology and evolution [7]. However, all of these studies have overlooked the micro-scale structure of the shell, in addition to its relationship with environmental pressure. 

In this context, we investigated how the land snail *N. hookeri* might adjust its biomineralised tissue to cope with heterogeneous environments. First, we focused on the shell micro-scale structure and its heterogeneity among the samples. We hypothesize that, in the event of calcium unavailability, a possible reduction in shell thickness may occur, as observed in marine prosobranchs [7] and opisthobranchs [11], with minimal modification to the shell micro-scale structure, but with an increase in the chitin ratio of the organic matrix [11]. In addition, we analysed variations in shell morphometrics (size, thickness and micro-scale structure) with respect to certain environmental conditions; namely, habitat, soil mineralogy and elevation. The formation of ecophenotypes based on shell morphometrics is proposed, with their distribution potentially being correlated with elevation and habitat typology, in addition to the availability of free exchangeable calcium in soil minerals.

## Material and Methods

### Ethic Statements

The French Polar Institute (IPEV) is the authority that supported this research based on the advice of its scientific council. The sites visited during this study did not require any access authorization.

All research and data reported here were obtained in compliance with all current French laws: no permission was required for collecting land snails in the French sub-Antarctic islands. In addition, the land snail *N. hookeri* is not an endangered or a protected species.

### Site and species

**Figure 1:**
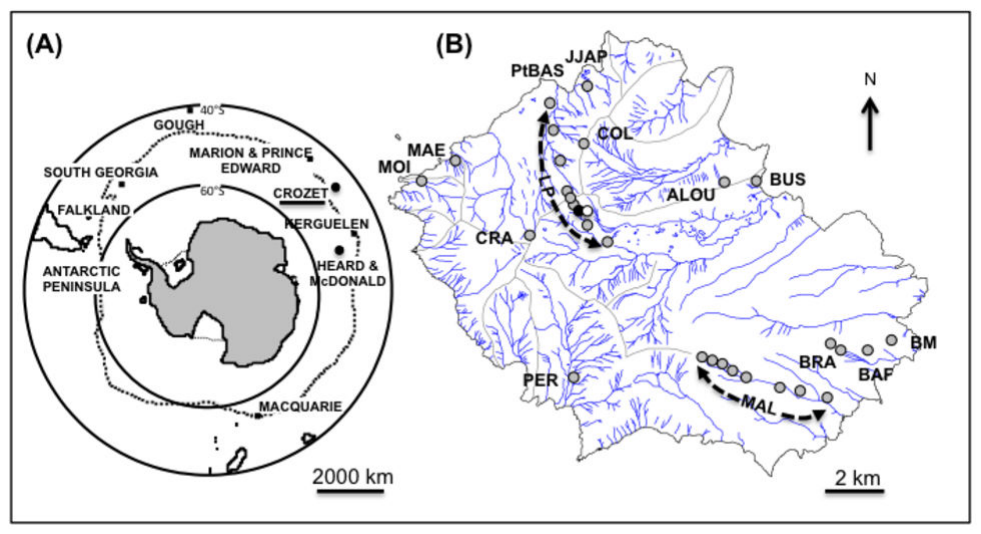
Sub-Antarctic Islands and study sites. Location of Crozet archipelago within the sub-Antarctic region (**A**), geographical location of the 30 sites sampled on Possession Island that belongs to Crozet archipelago (**B**). The sites are represented by dots and dotted lines along two altitudinal gradients: Labourage-Pâturage (LP, n = 9 sites), Malpassée (MAL, n = 8 sites). Other abbreviations on the map: ALOU = Crête de l’Alouette, BAF = Base Alfred Faure, BM = Baie du Marin, BRA = Mont Branca (200 and 300 m), BUS = Baie Américaine, COL = Col, CRA = Mont des Cratères, JJAP = Jardin Japonais, MAE = Mare Aux Éléphants, MOI = Moines, PER = Pérouse, PtBAS = Pointe Basse. The black and the white dots indicate the two habitat types, M1 and M2 respectively, that were sampled at LP 550 m (see Fig. 4).

The Crozet Archipelago falls within 45° 30ʹ and 46° 30ʹ S, 50° 00ʹ and 52° 30ʹ E of the sub-Antarctic region (Figure 1A) in the South Indian Ocean Province. Of the islands in the archipelago, Possession Island (46° 25ʹ S, 51° 45ʹ E, Figure 1B) is a strato-volcano characterised by a mountainous topography (Pic du Mascarin at 934 m) that extends across a very short distance (18 km long, 15 km wide, [17]). The topography of Possession Island is combined with a wet and windy climate, resulting in an estimated decrease in the temperature of about 0.8 °C for every 100 m of elevation [16]. The annual mean temperature at Base Alfred Faure is 5.3 °C, with monthly average temperatures ranging from 3.2 °C in winter to 8.1 °C in summer (Météo France 1970–2010 records). The volcanic history is complex; the most recent eruptions were ‘strombolian’ events (5000 to 10000 years BP) [17]. The composition of the lava is almost homogeneous, and the soil composition is mainly characterised by the presence of clay minerals that crystallise after the alteration of volcanic glasses and primary minerals of the lava. Soil diversity arises from the heterogeneous accumulation of clay minerals, depending on local climatic conditions (wind, rain and snow). Consequently, the calcium available in the soils is expected to vary from one site to another.

*Notodiscus hookeri* is the only native terrestrial gastropod species found in the South Indian Ocean islands and archipelagos (Crozet, Kerguelen, Heard, Marion and Prince Edward), and is limited to South Georgia in the South Atlantic Province [18]. *Notodiscus hookeri* is classified in the Charopidae, which is a family of litter-dwelling snails that are well represented in Australia. The biology of the species is poorly known. Shell growth does not stop on reaching sexual maturity, but decelerates considerably, with the biggest shells measuring 7.5–7.7 mm in size [20], [Charrier, unpublished results]. It is a gregarious species that lives under moist stones, moss and wet vegetation [18]; however, it is also widespread in fell-field areas, which are characterised by very low vegetation cover. Yet, the soil is known to be a nutrient resource for *N. hookeri*, since this species has been found to significantly increase calcium release in solutions derived from plant litter [21]. 

For this study, 30 sites on Possession Island were sampled, of which 17 sites were distributed along two altitudinal gradients; specifically Labourage-Pâturage (LP, 200–600 m north side, summit at LP 700m, 600–300 m south side) and Malpassée (MAL, 100–800 m) (Figure 1B). The 13 other sites were selected at random, depending on accessibility, and were situated on the east and west parts of the Labourage-Pâturage and Malpassée altitudinal transects. We surveyed the habitat heterogeneity of the fell-fields, the summits and the coastal plains of Possession Island.

### Variation in the morphology of the shell micro-scale structure

Snails were sampled during 2006, 2008 and 2009. Thirty sites were analysed, with just one sampling event per site being made (Figure 1B). We first defined cohorts within a population collected at Base Alfred Faure in 2006 (see SIF, Text S1). According to the methodology developed by Bhattacharya [22], we divided the sampled population into 0.25 mm size classes, to calculate their frequencies. Then, we resolved this distribution into its Gaussian components. From a sample of 210 snails, four size classes were delineated: hatchlings (< 2.0 mm), extremely difficult to find in the field, juveniles (≥ 2.0 and < 4.0 mm) and two cohorts of adult snails, called adults-1 (≥ 4.0 and < 5.0 mm) and adults-2 (≥ 5.0 mm) (see SIF, Figures S1A, S1B).

Principal component analysis based upon shell morphometrics was carried out on the snails sampled from the 30 sites (n = 330 snails). We delineated three different age classes, juveniles, adult-1 and adult-2 snails. All juveniles were clustered into one group. The two adult age classes were dissociated into two distinct groups (see SIF, Figure S2). We reasoned that adult snails represent well-developed and unambiguous shell characteristics, in particular the thicknesses of the shell layers. Therefore, this work focused on all of the adults; hence, the soft bodies of all individuals were dissected to check the maturity of the genital tract, and to validate the adult stage of the sampled snails. 

Eight individuals were used from each site, with each group containing four adult-1 snails and four adult-2 snails. Adult-2 snails were not collected from sites LPN 200, LPN 400, LPN 600, LPS600 and COL; therefore, the final number of snails analysed was n = 220. The last whorl of the shell was cut into separate pieces before coating with gold palladium (see SIF, Text S2). Variations in the shell micro-scale structure of the fractured surface (obtained by the mechanical breaking of the shells) were observed with a scanning electron microscope (SEM JEOL JSM 6301F). Measurements were made to determine the thicknesses of the organic layer (OL) and the mineralised layer (ML), in addition to the total shell thickness, including the periostracum (Thick). Three fractures per shell and three measurements per fracture were performed. A non-parametric Spearman test was used to explain variations in the OL/ML ratio (response) with respect to differences in shell thickness (independent variable). The Spearman test was preferred in this case, because a quotient variable is unlikely to be normally distributed. Computations were performed with R [R development core team 2013, version 3.0.2].

### Biochemical characteristics of the insoluble organic layer

Biochemical analyses were carried out using adult snails (n = 80) collected aseptically at BRA200, and then killed by heating at 60 °C. The organic layer was analysed by solid state nuclear magnetic resonance (NMR) analysis (n = 50 snails) and by the proteomic approach (n = 30 snails). The shells were mechanically broken, and the insoluble organic layer (OL) was separated with thin forceps from the mineral fraction under a stereomicroscope.

#### NMR analysis

Solid-state NMR analysis was applied to the OL of the snails (See SIF, Text S3). {^1^H}^13^C experiments under magic angle spinning (MAS) were performed on an Avance 300 Bruker spectrometer (7.1 T) operating at Larmor frequencies of 300 MHz and 75.4 MHz for ^1^H and ^13^C respectively [23,24]. Samples were packed on standard 4 mm rotors, and spun at 5 kHz at the magic angle. (^1^H π/2 pulse =5.8 µs, CP mixing time = 2 ms). The pure amino acids used for the OL characterisation of NMR peaks were identical to those detected by the proteomic analysis of the OL: Glycine, L-Isoleucine, L-Leucine and L-Valine. ß-Chitin from shrimp shells and amino acids were purchased from Sigma-Aldrich Chimie (St Quentin Fallavier, France). The spectra were processed using Dmfit software [24]. 

#### Proteomic approach

The OL membranes of snails collected aseptically were treated with 20 µg of trypsin (Proteomics grade, Sigma-Aldrich, St-Quentin Fallavier, france) overnight. The trypsin-digested material was further treated with 0.6% TFA [25] (CarloErba, Val de Reuil, France) in water containing 0.1 mM of Ditiothreitol (DTT). The suspension was incubated for 7 h at 65 °C, before the recovery of the supernatant. A second digestion of the TFA-insoluble layers was performed overnight in 6% TFA in water with 0.1 mM DTT at 65 °C. The assay for the peptide content in the TFA generated supernatant was completed using the bicinchoninic acid method. The peptides were separated on a C_18_ column (150 x 0.5 mm, Interchim, Montluçon, France) coupled to an ESI-QqTOF hybrid mass spectrometer (Pulsar, Applied Biosystems) using information dependent acquisition (IDA). After a 2 sec MS scan, the two most intense multiply charged precursor ions (+2 to +4) were selected for subsequent 2 sec MS/MS spectral acquisitions. The data were acquired and analysed with the Analyst QS software (v. 1.1). The minimum threshold intensity of the ion was set to 10 counts. The fragment ions obtained from the MS/MS peptides were searched for using an in-house version of the MASCOT search engine (Matrix Science, London, UK, v. 2.1) against the NCBInr protein database (March 2010, see SIF, Text S4). 

### Adjustment of shell micro-scale structure to heterogeneous environmental conditions

#### Habitat typology and altitude

The 30 sites that were investigated reflect the diversity of habitats originating from the oceanic volcano structure of Possession Island [17], and also reflect the climatic gradients of the island. The habitats were divided into five types: (1) grassy coastline dominated by Poaceae (*Poa cookii*, *Agrostis magellanica*) and fernbrakes (*Blechnum penna-marina*); (2) coastline dominated by vascular plants, including *Crassula moschata* and *Leptinella plumosa*, while the soil characteristics were influenced by bird droppings and salt spray. Peat cover tends to be an important factor determining where plants grow; (3) mesic fell-field situated above 150 m elevation, with bryophytes dominating in both species abundance and surface cover, because they form large balls. Lichens and vascular plants, including the sub-Antarctic cushion plant *Azorella selago* and the Kerguelen cabbage *Pringlea antiscorbutica*, accompany the bryophytes; (4) xeric fell-field situated above 400 m, where lichen occupancy is maximal; (5) xeric fell-field deprived of vegetation, which is characteristic of the mountain peaks (700–800 m) but also occurred at lower altitudes (500–600 m).

#### Soil mineralogy

Soil samples were collected from the ground surface of each site, where the snails were collected. Samples were dried overnight in an air-circulation oven (60 °C), and then crushed for 1 min to pulverize the clods. pH was determined using 20 g of the sample after agitation for 1 h with 50 mL of demineralised water. Dry samples (100 g per site) were used to determine the proportion of the soil particles according to their size as ≥2 mm, ≥1 mm, ≥0.5 mm, ≥0.2 mm, ≥0.1 mm and <0.1 mm. 

We looked for the presence of clay in each soil sample. For samples containing clay, we determined the occurrence of smectite, among other clay minerals. The presence and the nature of clay minerals in each soil sample were determined using X-Ray Diffraction [26,27] (see SIF, Text S5 and Figures S3, S4). Among these minerals, smectite is the only mineral that contains Ca, which occupies an exchangeable position in the mineral structure and is, thus, accessible to snails. Therefore, the presence of smectite was used as a qualitative independent variable.

#### Matching shell morphometrics with environmental variables

We used Redundancy Analysis (RDA) [28] as a multivariate approach to identify the environmental variables that best explained variations in shell morphometrics. This statistical method analyses the relationship between two tables recorded on the same set of individuals; the first table contains the multiple responses measured on each individual, the second table provides the candidate predictors, characterising generally the environment of each individual. Thus, the two tables have the same number of rows. Basically, the method includes two steps. First, all responses are predicted by a linear model through a complete set of independent variables. Second, the table of the predicted values is analysed by PCA. The response table (=shell parameters) was composed of the shell microstructure measurements (Thick, OL, ML), shell size (Size) and aperture diameter (Aper). The table of predictors was composed of two factors (habitat and soil mineralogy) and three numeric covariates (altitude, granulometry and pH). The habitat was classified into the five previously specified types; namely, (1) grassy coastline, (2) coastline with peat cover, (3) mesic fell-field, (4) xeric fell-field rich in lichens and (5) xeric fell-field deprived of vegetation. The soil analysis produced three ordered levels: (1) clay deprived of exchangeable calcium, (2) no clay and no calcium and (3) clay with Ca-smectite. This analysis was completed by a cluster analysis based on the scores obtained from each individual of the two first axes of the RDA. The distance between individuals was the ordinary Euclidian distance. The Ward method [29] was used as the clustering criterion. This analysis aimed to detect whether some natural classification emerged to form clusters. 

To assess the significance of the relationship of shell micro-scale structure with various environmental parameters, we performed the Monte-Carlo test with 1000 permutations based on the percentage of explained variance. The statistic of the Monte-Carlo test consists of the total inertia of the predictor analysis. A histogram is built for 1000 random permutations of the individuals between the columns of the morphometric table, providing 1000 inertia values. The observed total inertia of the actual analysis is then compared to the quantiles of the cumulative histogram. The total inertia is the sum of the eigenvalues of the second PCA that is produced on the predicted table. This crude test permits the global null hypothesis that the morphometric table has no statistical relation with the environmental table to be rejected. Afterwards, a multivariate analysis of variance (MANOVA) was used to test whether the shell parameters differed significantly among clusters. Since several individual were clustered in each site, the site was treated as a random factor for testing higher-level effects. Statistical analyses were performed with two R packages, the RDA analysis was carried out with Ade4 [30] and the Vegan package was used to evaluate the fraction of the variation that was accounted for by the environmental variables [31]. 

If environmental variation promoted different phenotypes with specific shell micro-scale structure traits in response to living conditions, then it would be necessary to investigate whether the mixing of two different age cohorts could artificially maximize microstructural differences, due to shell size increment during adult growth. Therefore, by employing analysis of covariance (ANCOVA), we looked for the relationship between shell size and shell thickness according to soil mineralogy, because shell growth is dependent on calcium dietary intake [32]. Furthermore, the causal relationship between shell thickness and growth rate in molluscs seems to be well supported, experimentally [33]. The best-fit model was determined according to the Akaike information criterion (AIC) value. To assess the strength of correlations in soils with Ca-smectite (Ca^+^) and soils without clay or Ca-smectite (Ca^-^), linear regressions from the ANCOVA summary were calculated between shell thickness and shell size (here, Ca-smectite containing soils are referred to as “calcic soils”).

The entire data set, including the response table (ad12.txt) and the table of predictors (Environnt.txt), is provided in the SIF, Text S6. The scripts used with R packages, related to RDA and MANOVA, are provided in the SIF, Text S7. The data set used for ANCOVA (Corr Size_Thick.txt) is provided in the SIF, Text S8.

## Results

### Occurrence of shell micro-scale structure phenotypes

**Figure 2:**
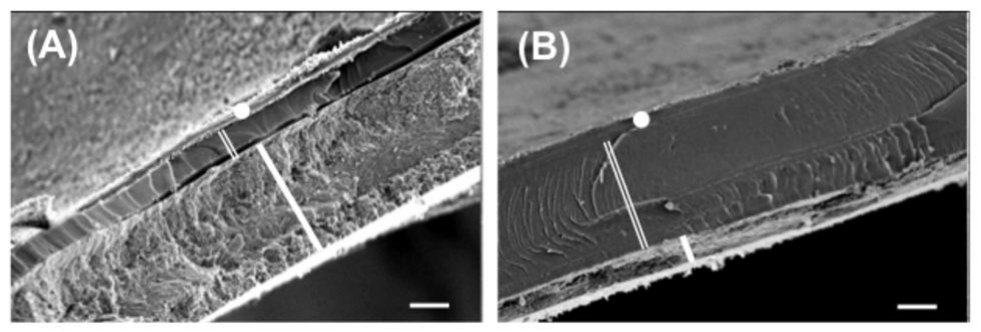
Scanning Electron Microscopy of the shell micro-scale structure of *Notodiscus hookeri*. The cross sections show a layered architecture of the shell and two contrasted phenotypes. The outer periostracum (full white circle), the innermost mineralised layer (ML, thick white line) and, in between, an organic layer (OL, double white line) (**A**, **B**), the OL/ML ratio may be reversed according to snail population, BUS (**A**) or BRA (**B**). Scale bars, 10 µm.

SEM observations of adult shells (n = 220 snails) revealed an unexpected shell micro-scale structure, due to the presence of a smooth organic layer (OL), rather than a fibrous layer. In addition, different phenotypes with OL/ML ratios ranging from 0.2 in hard shells (Figure 2A) to 5.4 for highly flexible shells (Figure 2B) were recorded. The organic layer clearly separated the periostracum from the mineralised layer (ML). A negative correlation was found between shell thickness and the OL/ML ratio (Spearman, n = 220, rho = - 0.522, p < 0.001).

### Organic layer of the *Notodiscus* shell

**Figure 3:**
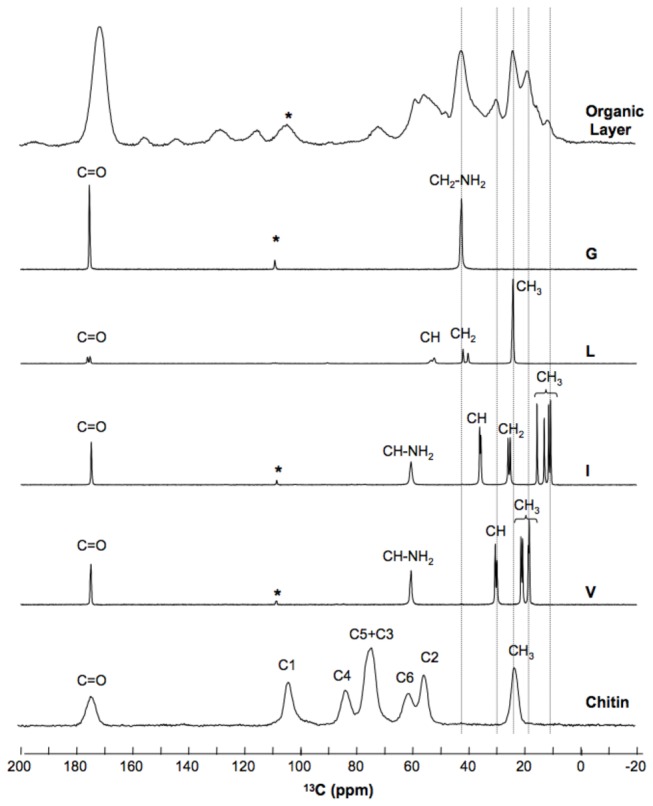
{^1^H}^13^C CPMAS spectrum of the organic layer of *Notodiscus hookeri* shells. The spectrum of the organic layer of shells originating from Branca site are compared to the spectra of ß-chitin powder and to the most abundant L-amino acids found in this layer (G = glycine, L = leucine, I = isoleucine and V = valine). The major peaks of the organic layer can be ascribed to each amino acid, as shown by the dotted lines. The chitin signature, indicated by the C1 to C6 carbon ions, is not detected in the organic layer. Asterisks (*) represent spinning sidebands.

Solid-state NMR spectroscopy of the OL confirmed the presence of glycine, leucine, isoleucine and valine in the organic layer, and revealed the absence of chitin (Figure 3). Solid-state NMR replicates of the OL layer of snails from both MAL800 and BRA produced the same profiles (See SIF, Text S3 and Figure S5). The proteomic approach identified glycine-rich proteins (GRPs) from the organic shell layer. The GRPs matched those found in insects (*Bombyx mori*, *Apis mellifera*, *Drosophila*), molluscs (*Pinctada fucata*) and plants (*Sorghum bicolor*) (see SIF, Text S4 and Table S1). Many of the peptides that matched with the proteins in the database contained either GG or GGG as the repeating motifs in their primary sequence, and were interrupted by isoleucine or leucine. 

### Matching shell morphometrics with environmental variables

**Figure 4:**
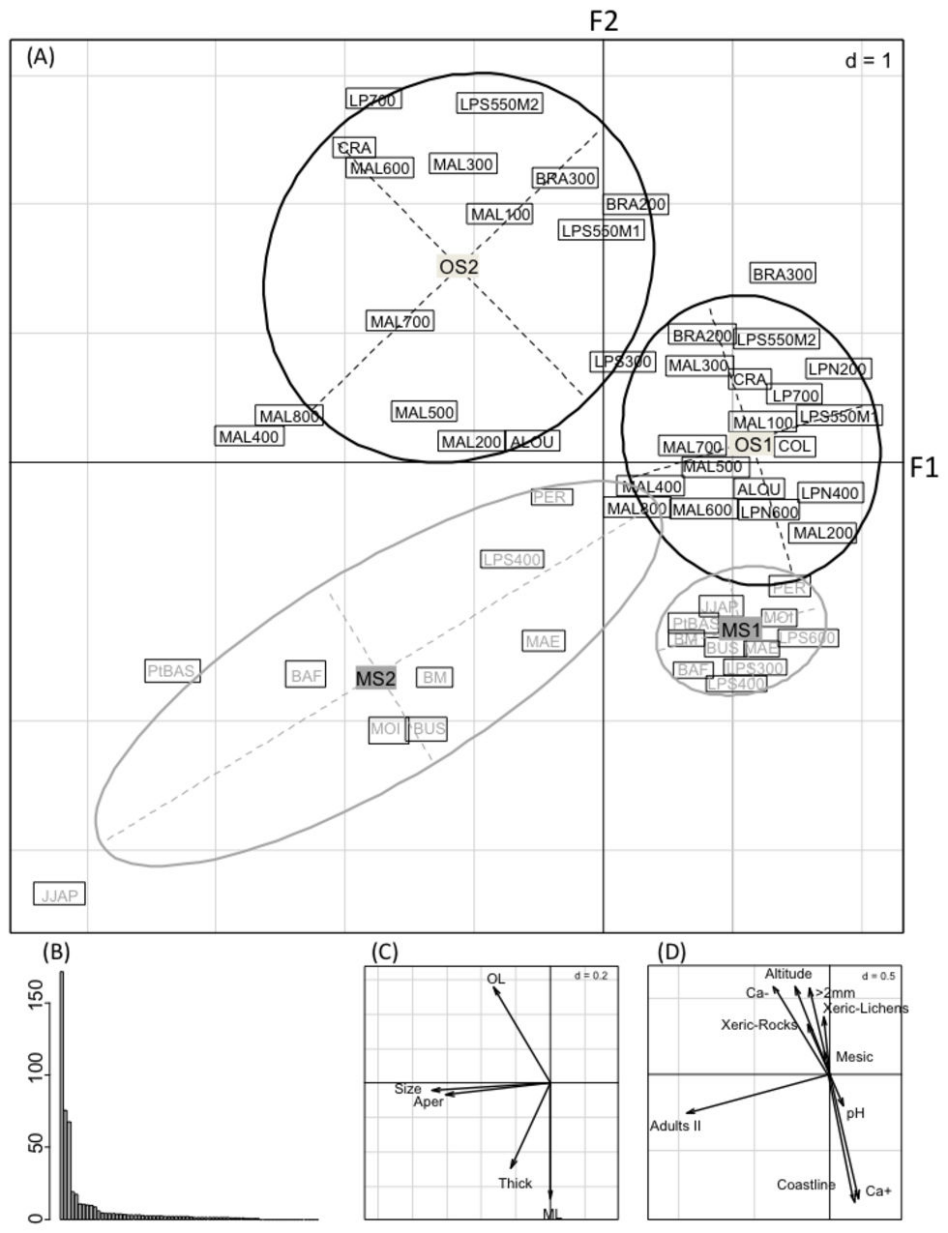
Redundancy analysis (RDA) of shell parameters matched with environmental variables in adult *Notodiscus hookeri*. Panel A shows the plot of the first two components axes of the RDA where the position of the sites is the gravity center of the sample (n=8 adult snails). The 95% confidence ellipses on the main graph illustrate a clear-cut separation of two ecophenotypes per adult age with a mineralised-shell (MS1 for adult-1, MS2 for adult-2) or an organic-shell (OS1, OS2). Panel B shows the distances between the individuals and the largest distances between the nodes were used to separate the clusters (panel A). No adult-2 snails were collected at the sites LPN 200, LPN 400, LPN 600, LPS600 and COL, therefore the number of snails analysed was n = 220. For abbreviations, see the Figure 1. Panel C is the projection of the response table (shell parameters) correlations with the RDA axes. Panel D is the projection of the environmental variables correlations with the RDA axes. Abbreviations of the shell parameters are: shell size (Size) and shell thickness (Thick), shell aperture diameter (Aper), thicknesses of the organic layer (OL) and the mineral layer (ML). Abbreviations of the sites along altitudinal gradients are: Malpassée (MAL from 100 to 800 m), Labourage-Pâturage (LP North (N) and South (S), from 200 to 600 m, two habitats (M1, M2 at LPS550 m), LP culminates at Mont 700 (LP700). Refer to Figure 1 for other abbreviations; Abbreviations of the soil types are: exchangeable calcium in clay (Ca+), soil deprived of clay and calcium (Ca-); Habitat typology is referred to as Coastline, Mesic, Xeric-rocks and Xeric-lichens; the fraction of particles > 2 mm in the soils is abbreviated: >2 mm.

RDA was performed on the shell morphometrics with respect to environmental variables (Figure 4). The first two-dimensional axes of the RDA extracted 97.60% of the total variance, of which 55.76 was on the first axis (F1) and 41.84 was on the second axis (F2). The first axis showed that shell growth had a strong effect with two correlated variables, shell size and aperture diameter. Environmental variables contributed significantly to variations in shell measurements among clusters, since they explained 62.54% of the total variance on the two first axes (Monte Carlo test, p < 0.001). Four clusters were delineated, and revealed bi-modal variation in the shell micro-scale structure that clearly separated each cohort of adult snails into two phenotypes explained by the environment, referred to as MS and OS ecophenotypes (Figure 4). The MS-ecophenotype was characterised by a thick and well-mineralised shell (MS), and was mainly found on the coastline. The MS-ecophenotype was possibly associated to the calcium availability from natural minerals (Ca-smectite) or from solutions leaching from the cemented roads found at the station (BAF) and surrounding area (Baie du Marin: BM). Conversely, the OS-ecophenotype was characterised by thin organic shells (OS), and might be correlated to the absence of Ca-smectite, and to a higher percentage (>40%) of large particles found in mesic and xeric fell-field soils at high altitudes, where lichens and mosses are the main trophic resources. There were significant differences among the four clusters with respect to each environmental variable (MANOVA, Table 1), including the overlap of habitats and altitudinal factors. In fact, the Akaike criterion value (6144.73), which is used as a measure to best fit the model, did not change when introducing altitude into the model with habitat typology. 

**Table 1:** Summary of MANOVA made on shell parameters to compare clusters after RDA.

Source of variation	df	Pillai Test	Approximate F	df	p
Particles > 2 mm	1	0.724	91.824	5	<0.001
Adult snail Cohort	3	1.836	55.859	15	<0.001
pH	1	0.421	25.427	5	<0.001
Soil	2	0.402	8.857	10	<0.001
Altitude	1	0.171	7.227	5	<0.001
Habitat	4	0.373	3.663	20	<0.001
Site:habitat	19	1.639	4.593	95	<0.001
Snail cohort:soil	3	0.295	3.856	15	<0.001
Snail cohort:habitat	5	0.457	3.603	25	<0.001
Snail cohort:habitat:soil	1	0.031	1.103	5	NS
Residuals	179				

A multivariate response variable was created by binding together the shell parameters then was used to test the significance of the environmental variables. Since individuals were clustered at each site (n = 8 individuals at n = 26 sites, n = 4 individuals at n = 4 sites), the site was treated as a random factor for testing the higher-level effects. No adult-2 snails were sampled at the four sites LPN 200, LPN 400, LPN 600, LPS600 and COL. For abbreviations, see the Figure 1.

### Relationship of shell size and thickness with calcium availability

**Figure 5:**
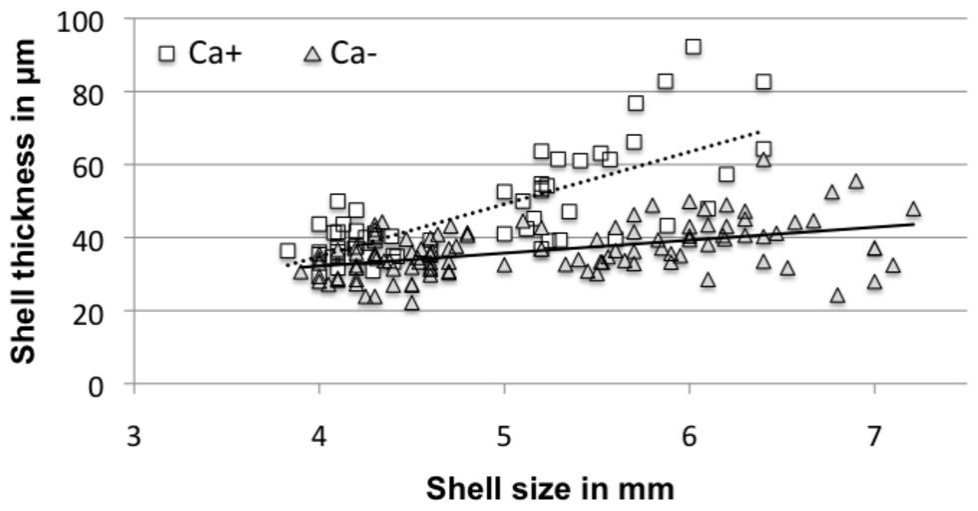
Linear regressions analysis using shell thickness and shell size in adult *Notodiscus hookeri*. Two linear regressions, calculated from ANCOVA summary (see SIF, Text S9), are represented as a function of availability of calcium in the soil. Ca+ (square), available calcium in clay, y = 15.08x-25.77; Ca- (triangle), soils deprived of calcium and clay, y=3.55x+17.98.

Soil mineralogy significantly influenced the correlation between shell size and shell thickness (ANCOVA, Table 2 and SIF, Text S8-S9). We focused on the significant positive correlations between shell size and shell thickness when calcium was available for snails (see SIF, Text S9; t = 10.642, ddl = 114, p < 0.001; Figure 5) and on soils deprived of clay and calcium (see SIF, t = 4.400, ddl = 114, p < 0.001; Figure 5). On soils deprived of calcium and clay, the biggest shells measured above 7.0 mm in size, whereas shell size did not exceed 6.5 mm for snails found on calcic soils (Figure 5). Linear regressions were used to calculate variations in shell thickness within the same shell size range (i.e. between 4.0–6.5 mm for each group). We noticed that shell thickness ranged from 35 µm to 41 µm on soil deprived of calcium and clay. Conversely, on calcic soils, shell thickness increased by more than a factor of two (from 35 µm to 72 µm). 

**Table 2:** ANCOVA performed on shell size (variable) and shell thickness (response) according to soil mineralogy (factor).

Source of variation	df	F value	P
DRXCa	2	36.403	<0.0001
DRXCa:Size	3	46.229	<0.0001
Residuals	214		

Abbreviations are: DRXCa for soil mineralogy and Size for shell size.

## Discussion

### Shell micro-scale structure and organic components

The shell micro-scale structure of *N. hookeri* represents a unique feature among gastropods, with it being characterised by a dense and homogeneous organic layer that is loosely attached to the upper periostracum and the inner mineral layer. 

Since the chitinous framework proposed in different models of shell calcification is subject to controversy [10,34], we compared the NMR spectra of the *Notodiscus* organic layer with that of pure chitin powder, and found no convergence. The absence of chitin has been previously reported in the slug shell *Ariolimax columbianus* [34], indicating a common feature with *N. hookeri*. However, the *N. hookeri* shell structure is a distinctive feature compared to other terrestrial slugs [35], in addition to opisthobranchs within the gastropods, in which the regressed shell is known to be chitinous [11]. 

The prevalence of glycine in the protein sequences is a characteristic of some shell matrix protein families, exhibiting homologies among molluscan species [36,37]. Glycine-rich domains have been hypothesised to contribute towards strengthening the cell wall in plants [38], and might provide flexibility and strength to the cuticle of insects [39]. Undoubtedly, the OS-ecophenotype, which is specific to *N. hookeri*, has a highly flexible shell. This feature could facilitate the sliding of the shell within small rock crevices, without causing injury to the body. However, higher protein shell synthesis might be costly for these snail populations, because shell thickness decreases at higher OL/ML ratios. In fact, Palmer [40] estimated that the soft shells of marine gastropods with high protein content are more expensive to produce compared to well-mineralised hard shells.

Slower body growth might be related to an increase in shell thickness [40,41]. In the case of *N. hookeri*, snails of the OS-ecophenotype are characterised by thin and large shells. This result indicates that these snails might exhibit a more rapid rate of body growth compared to snails belonging to the MS-ecopenotype, which have thick and small shells. Therefore, plasticity in the micro-scale structure might reflect the costs and benefits during both growth and reproduction, which involves eggshell formation. Our observations in this study indicate that calcium availability in the environment constrains the shell thickness of *N. hookeri*, and might influence calcium mobilisation by parents during egg laying [42].

### Environmental factors influencing shell micro-scale structure and shell size variation

In the present study, we analysed variation in phenotypic traits along spatial (elevation, habitat typology and soil mineralogy) and temporal (two adult cohorts) scales. Our investigation combined quantitative and qualitative analyses of trait variation with environmental factors. These factors accurately described (62.54% of the total variance) the occurrence of MS- and OS-ecophenotypes, and were also correlated with snail distributions at a microgeographic scale. 

Multivariate analysis showed that ML thickness was related to the availability of exchangeable calcium in soil minerals, and that the increase in shell thickness during growth was due to the widening of the mineral layer. It is well known that snails are able to detect inorganic compounds, including calcium carbonate present in the soil or plants, and balance their diet to optimize calcium intake [43]. Calcium is involved in a number of physiological functions (digestion, reproduction and defence) in snails and slugs, and is stored in different body compartments [32]. Therefore, the requirement of calcium by organisms and its availability in the soil might explain why the snails of the OS-ecophenotype exhibited a predominantly organic skeleton. Snails with large and thin shells might have lower skeleton masses per unit body weight compared to thick-shelled snails, due to the higher cost involved in protein production [41]. This phenomenon might also have direct consequences on shell geometry, such as whorl number (as reviewed by [44]), and might indirectly impact snail physiology (water loss regulation and cold tolerance). For example, Ansart et al. [45] demonstrated that adults of the land snail *Cornu aspersum* are larger compared to immature snails, and have a lower supercooling ability, surviving longer to exposure at -5 °C. 

Matching shell micro-scale structure with environmental variables led to another important finding; whereby, OL thickness is positively correlated with large soil particles and habitat typology, not just altitude. This finding indicates that the OS-ecophenotype is representative of mesic and xeric fell-fields found at high altitudes. Hence, the heterogeneous landscape of Possession Island might contribute towards determining shell ecophenotypes at a microgeographic scale, similar to the altitudinal factor. 

### *N. hookeri* as a model for studying evolutionary adaptation under environmental pressure

This study is the first to demonstrate that gastropod shell micro-scale structure responds to environmental heterogeneity leading to the formation of ecophenotypes. The snails exhibited bi-modal variation, represented by a thick mineralised shell versus a thin organic shell, which illustrates the presence of within-species microstructural phenotypic variation in *N. hookeri*. This pattern increased along a temporal scale, because adult-2 snails were more clearly separated into two clusters compared to adult-1 snails. This bi-modal variation also occurred along microenvironmental gradients, indicating that the scale of environmental variation, which was termed ‘grain size’ by [3], should be larger (i.e. in coarse-grained environments) compared to the scale of dispersal potential of *N. hookeri* over its lifetime. As noticed by [46], variations in phenotypic patterns represent necessary starting points towards understand the ecology and evolution of a species across geographical ranges. The special case of *N. hookeri* provides a good model because of its wide distribution in the sub-Antarctic region, as well as in the context of the hypothesis postulated by [19] stating that post-glacial dispersion by seabirds has facilitated the introduction of this species to several islands. According to this scenario, coastline introduction might represent the starting point of dispersion on Possession Island, with organisms of the MS-ecophenotype. The dispersion of the species in different environments, especially those deprived of free calcium, might have resulted in their taking advantage of the flexibility of traits associated with shell micro-scale structure. Moreover, OS-ecophenotypes have bigger shell sizes compared to MS-ecophenotypes, indicating an evolutionary trade-off between mineral thickness and shell size. At present, we do not have evidence to state that these differences in the shell micro-scale structure patterns induce differences in the fitness of the populations. Key steps in the future would be to test the adaptive value of the plasticity of this phenotype in response to spatial and temporal environmental variations. In addition, transplant experiments are being attempted to test the underlying genetic basis leading to the newly described ecophenotypes.

## Supporting Information

Text S1 **Population structure.** This includes snails (*n* = 210) taken at Base Alfred Faure (BAF, Figure 1) on the Possession Island between January and April 2006. Living snails were collected at random from ‘sampling unit areas’ that were defined as single stones of at least 15 x 15 cm, under which a thorough search for snails was conducted. Shell measurements (Figure S1a) were obtained using a micrometre under a stereomicroscope, and analysed with Spot Software (v. 4.6) to the nearest 0.001 mm. We divided the sampled population into 0.25 mm size classes to calculate their frequencies and we resolved this distribution into its Gaussian components (Bhattacharya CG (1967) A simple method of resolution of a distribution into Gaussian components. Biometrics 23: 115-135). Each mode corresponded to individual-age groups or cohorts defined by their mean shell size in mm ± standard deviation (Sd) and by their proportion in the population (Figure S1b).
(DOCX)

Text S2 **Micro-scale structure analysis of the shell.** For scanning observations, 50 to 100 individuals were collected at each site (*n* = 30), taking into account sampling unit areas and a sampling effort limited to 20 min. The geographic coordinates of all the samples were recorded with a hand held GPS (Garmin, eTrex). The samples were immediately fixed in 70% alcohol. After measuring shell size and aperture, the last whorl of eight adult snail shells (i.e. four from each size class) was extracted and dehydrated in acetone for 24 h. The whorls were CO_2_ critical point dried (BALZERS, CPD 010), and then cut carefully into separate pieces that were schematically drawn under the stereomicroscope prior to coating with gold-palladium (JEOL, JFC 1100). Observations of three distinct fractures per snail were made with a scanning electron microscope (JEOL JSM 6301F). The measurements were based upon the scale bar on each picture and were made with GraphicConverter software (2002–2005 Lemke Software GmbH, v. 5.6.2.).
(DOCX)

Text S3 **Solid-state Nuclear Magnetic Resonance (NMR) analysis of the organic layer.** The methodology applied to snails from BRA200 was replicated on snails from MAL800 site (n = 50) (Figure S3).
(DOCX)

Text S4 **Proteomic approach.** The partial peptides sequences obtained from the Organic layer of BRA200 snails were listed in the Table S1 searched against NCBInr protein database.
(DOCX)

Text S5 **Soil mineralogy.** Each soil sample was analysed for its mineralogical composition, especially the presence of clay minerals and their characteristics. After sieving, the presence and the characteristic of clay minerals in the < 0.1 mm fraction of the soil samples were determined using X-Ray Diffraction (XRD). The XRD patterns of non-oriented powders of the soils were obtained with a CPS 120 diffractometer (Co Kα radiation at 0.178897 nm, operated at 40 kV and 25 mA). Based on these patterns, the presence of clay minerals was highlighted by a broad peak at around 1.4 - 1.5 nm (Figure S4). The finest fraction of each sample (< 2 µm) was separated and prepared as oriented sections. Then, the sections were analysed with an INEL G3000 diffractometer (Cu Kα radiation at 0.15418 nm, operated at 30 kV and 40 mA) in the region of short angles to identify the clay minerals under air-dried condition (AD), after saturation with Ethylene-Glycol (EG) and after heating overnight at 550°C (H). The nature of the clay mineral was determined by the position of the (001) peak (Figure S5). Chlorite shows a peak at 1.4 nm on the AD-, EG- and H-pattern. Smectite is characterised by a peak at 1.5 nm on the AD-pattern, which shifts to 1.7 nm on the EG-pattern and to 1.0 nm on the H-pattern. Kaolinite is characterised by a peak at 0.7 nm on the AD- and EG-patterns, whereas no peak is observed on the H-pattern because being destroyed by heating. A peak attributed to illite or mica at 1.0 nm is present on the AD-, EG- and H-pattern.
(DOCX)

Text S6 **The whole data set, in txt format, used in RDA (response table = ad12.txt and table of predictors = Environnt.txt.).**
(DOCX)

Text S7 **Scripts used for the RDA and for the MANOVA after RDA.**
(DOCX)

Text S8 **Data set (Corr Size_Thick.txt) used to carry out ANCOVA.**
(DOCX)

Text S9 **Summary of ANCOVA performed on shell size and shell thickness according to soil mineralogy.**
(DOCX)

Figure S1 **Features of shell measurements performed on the snail *Notodiscus hookeri*.** Size = the widest diagonal of the shell; Aperture = the widest diagonal of the shell aperture. The dotted part is the last whorl used to study shell micro-scale structure according to age (**A**) and cohorts of the snail *Notodiscus hookeri* during the summer recruitment period (January–April, n = 210), from a population collected in 2006 at Base Alfred Faure on Possession Island (**B**). Size classes are defined by shell size measurements. Four cohorts were delineated, based on shell sizes (mean in mm ± Sd) and relative contribution (in %) to the snail population: Hatchlings: 1.38 ± 0.21 (12 %); Juveniles: 2.90 ± 0.40 (28 %); Adults 1 (young): 3.96 ± 0.40 (37 %) and Adults 2 (older): 4.98 ± 0.38 (23 %).
(TIF)

Figure S2 **Principal Component Analysis (PCA) of shell parameters in both juveniles and adults of *Notodiscus hookeri*.** Four snails were sampled per site (n = 30 sites), but no juveniles were collected at the sites LP700 and CRA and no adult-2 snails were collected at the sites LPN 200, LPN 400, LPN 600, LPS600 and COL. Therefore the number of snails analysed was n = 332. The two first components axes of the PCA are presented. The shell parameters are symbolized by arrows and are superimposed to the individuals J (juveniles), Ad1 (adult-1) and Ad2 (adult-2). The 95% confidence ellipses on the graph illustrate the three cohorts where each individual is represented by a dot. In support to our argumentation in the main text, we can see that Juveniles are altogether in one group. The ecophenotypes make discrete clusters in adult-1 snails and are clearly delineated in adult-2 snails. Therefore, the differentiation in two ecophenotypes appears progressively with age. For abbreviations, see the Figures 1 and 4.
(TIF)

Figure S3 **X ray diffraction powder patterns of the < 0.1 mm fractions in soils**. The samples are from "Mare Aux Éléphants" and "Labourage Pâturage Sud, 550 m M2". The rectangular dotted line indicates the region of interest for the investigation of clay minerals. Other peaks (> 15 °2θ) are attributed to feldspars, magnetite and mica.
(TIF)

Figure S4 **X ray diffraction oriented patterns of the <2 µm fractions in soils.** The sample from "LabouragePâturageSud, 600 m Sud" is given as an example. -AD: air dried conditions, -EG: saturated with ethylene glycol, -H: heated to 550 °C.
Structural formulae are:.
for Ca-rich smectite (Ca-Sm),.
for Chlorite,.
*Si_4_(Al_2-x_R^2+^_x_*)*Ca*_*^x^/_2_*_ *O*_*10*_(*OH*)_*2*_ *nH*_2_ *O* for CA-rich smectite(Ca-Sm),
(*Si_4-x_Al_x_*)(*R^2+^_3_)*(*R^3+^_x_R^2+^_3-x_*)*O*_10_(*OH*)_8_ for Chlorite,
(*Si_4-x_Al_x_*)*Al_2_K_x_O_10_*(*OH*)_2_ for Illite and
*Si*_*2*_ *Al*_*2*_ *O*_*5*_(*OH*)_*4*_ for Kaolinite.
R^2+^ = Mg^2+^, Fe^2+^, Mn^2+^; R^3+^ = Al^3+^, Fe3.
(TIF)

Figure S5 **{^1^H}^13^C CPMAS spectrum of the organic layer of *Notodiscus hookeri* shells.** Shells originated from Malpassée (MAL) were compared with those from Branca (BRA) and put in relation to the spectra of ß-chitin powder and of the most abundant L-amino acids found in this layer (G = glycine, L = leucine, I = isoleucine and V = valine). For other explanations, see Figure 3.
(TIF)

Table S1 (DOCX
