# Correction: Soil Calcium Availability Influences Shell Ecophenotype Formation in the Sub-Antarctic Land Snail, *Notodiscus hookeri*

**DOI:** 10.1371/journal.pone.0092541

**Published:** 2014-03-10

**Authors:** The PLOS ONE Staff

Notice of Republication.

This article was republished on February 21, 2014 due to missing figures in the PDF. Please download this article again to view the figures.
