# Supplementary material for: Soil Calcium Availability Influences Shell Ecophenotype Formation in the Sub-Antarctic Land Snail, Notodiscus hookeri
Source: PLoS One. 2013 Dec 20;8(12):e84527. doi: 10.1371/journal.pone.0084527 (PMC3869943; doi:10.1371/journal.pone.0084527)
Supplement: Text S1 — Population structure. This includes snails (n = 210) taken at Base Alfred Faure (BAF, Figure 1) on the Possession Island between January and April 2006. Living snails were collected at random from ‘sampling unit areas’ that were defined as single stones of at least 15 x 15 cm, under which a thorough search for snails was conducted. Shell measurements (Figure S1a) were obtained using a micrometre under a stereomicroscope, and analysed with Spot Software (v. 4.6) to the nearest 0.001 mm. We divided the sampled population into 0.25 mm size classes to calculate their frequencies and we resolved this distribution into its Gaussian components (Bhattacharya CG (1967) A simple method of resolution of a distribution into Gaussian components. Biometrics 23: 115-135). Each mode corresponded to individual-age groups or cohorts defined by their mean shell size in mm ± standard deviation (Sd) and by their proportion in the population (Figure S1b). (DOCX) [file pone.0084527.s001.docx]

**Text S1.**

**Population structure.** This includes snails (*n =* 210) taken at Base Alfred Faure (BAF, Fig. 1) on the Possession Island between January and April 2006. Living snails were collected at random from ‘sampling unit areas’ that were defined as single stones of at least 15 x 15 cm, under which a thorough search for snails was conducted. Shell measurements (Figure S1a) were obtained using a micrometre under a stereomicroscope, and analysed with Spot Software (v. 4.6) to the nearest 0.001 mm. We divided the sampled population into 0.25 mm size classes to calculate their frequencies and we resolved this distribution into its Gaussian components (Bhattacharya CG (1967) A simple method of resolution of a distribution into Gaussian components. Biometrics 23: 115-135). Each mode corresponded to individual-age groups or cohorts defined by their mean shell size in mm ± standard deviation (Sd) and by their proportion in the population (Figure S1b).
