# Supplementary material for: Soil Calcium Availability Influences Shell Ecophenotype Formation in the Sub-Antarctic Land Snail, Notodiscus hookeri
Source: PLoS One. 2013 Dec 20;8(12):e84527. doi: 10.1371/journal.pone.0084527 (PMC3869943; doi:10.1371/journal.pone.0084527)
Supplement: Text S2 — Micro-scale structure analysis of the shell. For scanning observations, 50 to 100 individuals were collected at each site (n = 30), taking into account sampling unit areas and a sampling effort limited to 20 min. The geographic coordinates of all the samples were recorded with a hand held GPS (Garmin, eTrex). The samples were immediately fixed in 70% alcohol. After measuring shell size and aperture, the last whorl of eight adult snail shells (i.e. four from each size class) was extracted and dehydrated in acetone for 24 h. The whorls were CO2 critical point dried (BALZERS, CPD 010), and then cut carefully into separate pieces that were schematically drawn under the stereomicroscope prior to coating with gold-palladium (JEOL, JFC 1100). Observations of three distinct fractures per snail were made with a scanning electron microscope (JEOL JSM 6301F). The measurements were based upon the scale bar on each picture and were made with GraphicConverter software (2002–2005 Lemke Software GmbH, v. 5.6.2.). (DOCX) [file pone.0084527.s002.docx]

**Text S2.**

**Micro-scale structure analysis of the shell.** For scanning observations, 50 to 100 individuals were collected at each site (*n* = 30), taking into account sampling unit areas and a sampling effort limited to 20 min. The geographic coordinates of all the samples were recorded with a hand held GPS (Garmin, eTrex). The samples were immediately fixed in 70% alcohol. After measuring shell size and aperture, the last whorl of eight adult snail shells (i.e. four from each size class) was extracted and dehydrated in acetone for 24 h. The whorls were CO_2_ critical point dried (BALZERS, CPD 010), and then cut carefully into separate pieces that were schematically drawn under the stereomicroscope prior to coating with gold-palladium (JEOL, JFC 1100). Observations of three distinct fractures per snail were made with a scanning electron microscope (JEOL JSM 6301F). The measurements were based upon the scale bar on each picture and were made with GraphicConverter software (2002–2005 Lemke Software GmbH, v. 5.6.2.).
