# Supplementary material for: Soil Calcium Availability Influences Shell Ecophenotype Formation in the Sub-Antarctic Land Snail, Notodiscus hookeri
Source: PLoS One. 2013 Dec 20;8(12):e84527. doi: 10.1371/journal.pone.0084527 (PMC3869943; doi:10.1371/journal.pone.0084527)
Supplement: Text S3 — Solid-state Nuclear Magnetic Resonance (NMR) analysis of the organic layer. The methodology applied to snails from BRA200 was replicated on snails from MAL800 site (n = 50) (Figure S3). (DOCX) [file pone.0084527.s003.docx]

**Text S3.**

**Solid-state Nuclear Magnetic Resonance (NMR) analysis of the organic layer.**

The methodology applied to snails from BRA200 was replicated on snails from MAL800 site (n = 50) (Figure S3).
