# Supplementary material for: Soil Calcium Availability Influences Shell Ecophenotype Formation in the Sub-Antarctic Land Snail, Notodiscus hookeri
Source: PLoS One. 2013 Dec 20;8(12):e84527. doi: 10.1371/journal.pone.0084527 (PMC3869943; doi:10.1371/journal.pone.0084527)
Supplement: Text S4 — Proteomic approach. The partial peptides sequences obtained from the Organic layer of BRA200 snails were listed in the Table S1 searched against NCBInr protein database. (DOCX) [file pone.0084527.s004.docx]

**Text S4.**

**Proteomic approach.** The partial peptides sequences obtained from the Organic layer of BRA200 snails were listed in the Table S1 searched against NCBInr protein database.
