# Supplementary material for: Soil Calcium Availability Influences Shell Ecophenotype Formation in the Sub-Antarctic Land Snail, Notodiscus hookeri
Source: PLoS One. 2013 Dec 20;8(12):e84527. doi: 10.1371/journal.pone.0084527 (PMC3869943; doi:10.1371/journal.pone.0084527)
Supplement: Text S5 — Soil mineralogy. Each soil sample was analysed for its mineralogical composition, especially the presence of clay minerals and their characteristics. After sieving, the presence and the characteristic of clay minerals in the < 0.1 mm fraction of the soil samples were determined using X-Ray Diffraction (XRD). The XRD patterns of non-oriented powders of the soils were obtained with a CPS 120 diffractometer (Co Kα radiation at 0.178897 nm, operated at 40 kV and 25 mA). Based on these patterns, the presence of clay minerals was highlighted by a broad peak at around 1.4 - 1.5 nm (Figure S4). The finest fraction of each sample (< 2 µm) was separated and prepared as oriented sections. Then, the sections were analysed with an INEL G3000 diffractometer (Cu Kα radiation at 0.15418 nm, operated at 30 kV and 40 mA) in the region of short angles to identify the clay minerals under air-dried condition (AD), after saturation with Ethylene-Glycol (EG) and after heating overnight at 550°C (H). The nature of the clay mineral was determined by the position of the (001) peak (Figure S5). Chlorite shows a peak at 1.4 nm on the AD-, EG- and H-pattern. Smectite is characterised by a peak at 1.5 nm on the AD-pattern, which shifts to 1.7 nm on the EG-pattern and to 1.0 nm on the H-pattern. Kaolinite is characterised by a peak at 0.7 nm on the AD- and EG-patterns, whereas no peak is observed on the H-pattern because being destroyed by heating. A peak attributed to illite or mica at 1.0 nm is present on the AD-, EG- and H-pattern. (DOCX) [file pone.0084527.s005.docx]

**Text S5.**

**Soil mineralogy.** Each soil sample was analysed for its mineralogical composition, especially the presence of clay minerals and their characteristics. After sieving, the presence and the characteristic of clay minerals in the < 0.1 mm fraction of the soil samples were determined using X-Ray Diffraction (XRD). The XRD patterns of non-oriented powders of the soils were obtained with a CPS 120 diffractometer (Co Kα radiation at 0.178897 nm, operated at 40 kV and 25 mA). Based on these patterns, the presence of clay minerals was highlighted by a broad peak at around 1.4 – 1.5 nm (Figure S4). The finest fraction of each sample (< 2 µm) was separated and prepared as oriented sections. Then, the sections were analysed with an INEL G3000 diffractometer (Cu Kα radiation at 0.15418 nm, operated at 30 kV and 40 mA) in the region of short angles to identify the clay minerals under air-dried condition (AD), after saturation with Ethylene-Glycol (EG) and after heating overnight at 550°C (H). The nature of the clay mineral was determined by the position of the (001) peak (Figure S5). Chlorite shows a peak at 1.4 nm on the AD-, EG- and H-pattern. Smectite is characterised by a peak at 1.5 nm on the AD-pattern, which shifts to 1.7 nm on the EG-pattern and to 1.0 nm on the H-pattern. Kaolinite is characterised by a peak at 0.7 nm on the AD- and EG-patterns, whereas no peak is observed on the H-pattern because being destroyed by heating. A peak attributed to illite or mica at 1.0 nm is present on the AD-, EG- and H-pattern.
