# Supplementary material for: Soil Calcium Availability Influences Shell Ecophenotype Formation in the Sub-Antarctic Land Snail, Notodiscus hookeri
Source: PLoS One. 2013 Dec 20;8(12):e84527. doi: 10.1371/journal.pone.0084527 (PMC3869943; doi:10.1371/journal.pone.0084527)
Supplement: Text S6 — The whole data set, in txt format, used in RDA (response table = ad12.txt and table of predictors = Environnt.txt.). (DOCX) [file pone.0084527.s006.docx]

**Text. S6: The whole data set, in txt format, used in RDA (response table = ad12.txt and table of predictors = Environnt.txt.)**

**Ad12.txt:**

SITE indiv OL ML Thick Size Aper Age

MAL100 1 16.65 14.01 31.24 4.6 2.4 Ad1

MAL100 2 16.14 17.6 35.95 4.5 2.3 Ad1

MAL100 3 12.56 13.66 27.2 4.5 2.2 Ad1

MAL100 4 12.64 14.37 28.33 4.1 2.3 Ad1

MAL200 1 10.17 25.53 37.41 4.2 2 Ad1

MAL200 2 15.14 15.39 32.31 4.2 2.1 Ad1

MAL200 3 12.49 14.46 28.87 4.1 2.1 Ad1

MAL200 4 8.81 17.42 27.98 4 2.1 Ad1

MAL300 1 16.44 17.81 35.63 4.3 2.4 Ad1

MAL300 2 20.19 10.39 31.81 4.6 2.5 Ad1

MAL300 3 17.38 11.51 30.35 4.7 2.5 Ad1

MAL300 4 21.79 11.99 35.25 4.6 2.5 Ad1

MAL400 1 15.05 25.96 43.16 4.71 2.43 Ad1

MAL400 2 15.13 24.47 40.86 4.64 2.48 Ad1

MAL400 3 17.57 17.95 37.67 4.74 2.46 Ad1

MAL400 4 13.31 20.96 35.76 4.2 2.3 Ad1

MAL500 1 16.98 24.68 43.49 4.3 2.3 Ad1

MAL500 2 21.75 20.58 44.28 4.34 2.4 Ad1

MAL500 3 15.52 16.33 33.61 4.35 2.2 Ad1

MAL500 4 11.26 11.95 23.91 4.25 2.15 Ad1

MAL600 1 15.13 24.19 41.19 4.15 1.9 Ad1

MAL600 2 19.52 25.47 46.78 4.36 2.55 Ad1

MAL600 3 16.01 19.84 38.02 4.09 2.12 Ad1

MAL600 4 17.58 19.62 38.36 4.43 2.31 Ad1

MAL700 1 12.91 18.93 34.21 4 2.2 Ad1

MAL700 2 17.33 17.18 36.83 4.55 2.43 Ad1

MAL700 3 18.66 20.88 40.62 4.8 2.5 Ad1

MAL700 4 14.71 16.87 33.55 4.38 2.41 Ad1

MAL800 1 13.9 19.86 34.53 4.53 2.6 Ad1

MAL800 2 13.18 22.06 36.92 4.7 2.7 Ad1

MAL800 3 12.35 25.9 39.55 4.47 2.49 Ad1

MAL800 4 11.17 27.8 40.95 4.3 2.43 Ad1

LPN200 1 21.76 10.3 33.45 4.1 2 Ad1

LPN200 2 23.36 8.01 32.04 4.6 2.4 Ad1

LPN200 3 15.09 10.16 25.82 4 2 Ad1

LPN200 4 16.99 5.76 23.35 4.1 2.1 Ad1

LPN400 1 7.43 15.01 23.1 4 1.7 Ad1

LPN400 2 13.82 15.76 30.5 4.2 2 Ad1

LPN400 3 18.36 17.2 36.34 4.7 2.5 Ad1

LPN400 4 13.77 18.23 33.66 4 1.9 Ad1

LPN600 1 13.88 20.7 35.67 4.3 2 Ad1

LPN600 2 15.32 16.85 33.51 4.8 2.5 Ad1

LPN600 3 8.86 25.79 35.65 4 2 Ad1

LPN600 4 14.01 16.26 31.26 4.61 2.1 Ad1

LP700 1 27.38 12.55 42.18 4.3 2.4 Ad1

LP700 2 18.28 11.44 31.67 4.2 2.1 Ad1

LP700 3 16.61 11.01 29.35 4 2 Ad1

LP700 4 16.26 11.17 28.69 4.1 2.3 Ad1

LPS600 1 7.02 27.89 35.99 4 1.8 Ad1

LPS600 2 8.88 19.42 29.32 4 1.9 Ad1

LPS600 3 8.68 25.7 34.94 4.1 2.1 Ad1

LPS600 4 9.28 31.02 41.83 4.2 2 Ad1

LPS550M1 1 17.44 10.5 29.73 4.5 2.5 Ad1

LPS550M1 2 16.32 10.7 28.58 4.3 2.2 Ad1

LPS550M1 3 16.45 11.92 29.86 4.1 1.9 Ad1

LPS550M1 4 13.96 11.28 26.01 4 1.9 Ad1

LPS550M2 1 21.44 10.34 33.14 4.6 2.3 Ad1

LPS550M2 2 20.14 9.12 30.78 4.7 2.3 Ad1

LPS550M2 3 20.01 8.56 29.78 4.6 2.4 Ad1

LPS550M2 4 18.97 7.18 27.29 4.2 2.1 Ad1

LPS400 1 6.88 27.81 36.1 4.1 1.9 Ad1

LPS400 2 6.99 29.31 37.1 4.1 1.9 Ad1

LPS400 3 6.13 39.49 47.51 4.2 2.2 Ad1

LPS400 4 8.06 29.36 39.27 4.6 2.1 Ad1

LPS300 1 8.28 32.15 41.94 4.1 1.8 Ad1

LPS300 2 9.99 33.05 44.78 4 1.9 Ad1

LPS300 3 10.08 33.56 45.08 4.2 2.1 Ad1

LPS300 4 10.31 26.12 38.06 4.1 1.8 Ad1

ALOU 1 14.31 17.38 33.12 4.6 2.4 Ad1

ALOU 2 11.72 22.13 34.81 4.3 2.4 Ad1

ALOU 3 10.69 16.94 28.49 4.2 2 Ad1

ALOU 4 13.16 16.68 31.44 4.4 2.2 Ad1

BRA200 1 25.5 9.66 36.16 4.61 2.62 Ad1

BRA200 2 24.67 12.75 39.86 4.6 2.5 Ad1

BRA200 3 21.54 12.51 34.28 4.31 2.32 Ad1

BRA200 4 18.67 7.45 27.19 4.05 2.1 Ad1

BRA300 1 28.05 4.81 33.3 4.7 2.1 Ad1

BRA300 2 27.17 5.21 33.33 4.54 2.34 Ad1

BRA300 3 25.83 5.22 31.86 4.5 2.25 Ad1

BRA300 4 18.46 3.82 23.88 4.3 2.2 Ad1

COL 1 19.97 18.86 40.01 4.3 2 Ad1

COL 2 19.28 14.67 34.41 4.6 2.1 Ad1

COL 3 14.6 9.51 25.24 4.2 1.9 Ad1

COL 4 14.62 16.44 34.31 4.3 2.1 Ad1

CRA 1 22.46 12.22 35.74 4.6 2.5 Ad1

CRA 2 12.58 13.29 26.99 4.4 2.4 Ad1

CRA 3 14.74 11.28 26.94 4.5 2.43 Ad1

CRA 4 12.44 8.74 22.2 4.5 2.4 Ad1

BAF 1 9.05 38.61 49.96 4.1 2 Ad1

BAF 2 9.78 29.26 41.5 4.1 2.12 Ad1

BAF 3 11.3 30.58 43.66 4.13 2.25 Ad1

BAF 4 8.79 30.44 41.33 4.08 1.91 Ad1

BM 1 8.2 26.08 35.33 4 2.1 Ad1

BM 2 10.27 29.34 41.16 4.3 2.3 Ad1

BM 3 10.37 28.2 40.32 4.3 2.4 Ad1

BM 4 8.83 33.06 43.7 4 2.1 Ad1

BUS 1 7.86 30.05 39.49 4.4 2.4 Ad1

BUS 2 4.56 29.49 35.46 4.1 2 Ad1

BUS 3 8.26 29.06 37.82 4.2 2.1 Ad1

BUS 4 7.94 27.71 36.72 4.4 2.2 Ad1

JJAP 1 7.74 27.16 37 4.19 2.27 Ad1

JJAP 2 7.23 25.88 34.62 4.26 2.2 Ad1

JJAP 3 6.31 26.67 34.63 4.13 2.39 Ad1

JJAP 4 11.09 27.32 40.33 4.39 2.18 Ad1

MAE 1 4.14 31.42 37.63 4.2 2.12 Ad1

MAE 2 5.19 23.9 30.83 4.29 2.16 Ad1

MAE 3 5.55 27.42 34.82 4.42 2.21 Ad1

MAE 4 5.73 24.21 31.67 4.1 2.3 Ad1

MOI 1 6.19 28.73 36.37 3.83 2.02 Ad1

MOI 2 9.97 27.67 39.08 4.3 2.1 Ad1

MOI 3 9.9 28.25 39.98 4.25 2.17 Ad1

MOI 4 9.52 23.4 34.67 4.07 2.01 Ad1

PER 1 9.09 26.13 36.63 4.1 2.3 Ad1

PER 2 6.86 22.4 30.64 3.9 2 Ad1

PER 3 10.59 21.09 33.03 4 2.2 Ad1

PER 4 10.76 23.93 35.52 4 2 Ad1

PtBas 1 5.4 28.36 35.06 4.4 2.3 Ad1

PtBas 2 4.4 27.57 33.34 4.4 2.3 Ad1

PtBas 3 7.82 26.81 35.59 4.6 2.4 Ad1

PtBas 4 6.54 27.68 39.8 4.3 2.2 Ad1

MAL100 1 29.06 8.22 40.28 6.4 3 Ad2

MAL100 2 26.71 10.6 39.43 5.5 2.6 Ad2

MAL100 3 23.36 10.76 35.62 5.9 2.8 Ad2

MAL100 4 19.78 9.07 30.15 5.5 2.7 Ad2

MAL200 1 14.87 32.13 49.86 6 2.9 Ad2

MAL200 2 12.45 22.05 36.2 5.7 2.7 Ad2

MAL200 3 16.24 24.49 41.56 5.7 2.7 Ad2

MAL200 4 12.5 22.86 36.87 5.2 2.8 Ad2

MAL300 1 22.9 8.24 33.26 5.9 2.7 Ad2

MAL300 2 34.08 8.27 43.43 6.1 2.8 Ad2

MAL300 3 29.06 9.11 39.44 6 2.8 Ad2

MAL300 4 30.14 8.09 40.42 6 2.8 Ad2

MAL400 1 11.68 35.93 52.52 6.77 3.2 Ad2

MAL400 2 16.15 26.13 45.06 6.3 3.1 Ad2

MAL400 3 13.16 25.85 40.58 6.18 3.01 Ad2

MAL400 4 15.3 36.41 55.5 6.9 3.4 Ad2

MAL500 1 17.11 30.24 48.94 6.2 2.8 Ad2

MAL500 2 14.97 23.29 39.38 5.83 2.8 Ad2

MAL500 3 15.86 28.39 46.27 5.7 2.7 Ad2

MAL500 4 25.79 21.34 48.86 5.8 2.6 Ad2

MAL600 1 35.8 10.38 48.08 6.54 3.42 Ad2

MAL600 2 23.02 11.24 36.91 6.28 3 Ad2

MAL600 3 24.46 14.09 40.66 6.6 3.2 Ad2

MAL600 4 25 8.29 36.49 6.1 2.72 Ad2

MAL700 1 19.62 19.54 42.99 6 3 Ad2

MAL700 2 18.82 26.21 47.8 6.08 3.15 Ad2

MAL700 3 17.41 17.7 39.68 6.19 3.07 Ad2

MAL700 4 18.82 14.12 35.06 5.95 2.79 Ad2

MAL800 1 10.79 35.56 47.93 7.21 3.19 Ad2

MAL800 2 11.2 32.85 44.6 6.67 2.96 Ad2

MAL800 3 12.42 28.22 41.19 6.47 3 Ad2

MAL800 4 16.39 25.49 44.2 6.57 3.07 Ad2

LP700 1 29.55 6.71 37.19 7 3.2 Ad2

LP700 2 20.2 6.44 27.97 7 3.3 Ad2

LP700 3 25.88 8.12 37.02 7 3.3 Ad2

LP700 4 17.74 5.59 24.27 6.8 3 Ad2

LPS550M1 1 21.69 5.95 28.4 5.7 2.8 Ad2

LPS550M1 2 23.26 7.16 30.86 5.4 2.6 Ad2

LPS550M1 3 22.63 9.58 33.22 5.6 2.5 Ad2

LPS550M1 4 24.41 8.65 33.94 5.4 2.6 Ad2

LPS550M2 1 28.1 3.56 32.44 7.1 2.8 Ad2

LPS550M2 2 29.63 2.77 33.5 6.4 2.7 Ad2

LPS550M2 3 24.44 3.75 28.53 6.1 2.8 Ad2

LPS550M2 4 28.04 2.7 31.73 6.53 2.7 Ad2

LPS400 1 7.36 31.17 39.52 5.6 2.7 Ad2

LPS400 2 7.3 28.07 36.89 5.2 2.4 Ad2

LPS400 3 7.33 34.09 43.28 5.88 2.9 Ad2

LPS400 4 6.42 31.49 39.31 5.3 2.6 Ad2

LPS300 1 16.37 18.08 34.78 5.3 2.5 Ad2

LPS300 2 19.06 15.28 35.83 5.3 2.5 Ad2

LPS300 3 23.6 16.09 42.5 5.3 2.2 Ad2

LPS300 4 19.71 12.85 34.83 5.2 2.4 Ad2

ALOU 1 15.34 26.04 42.76 5.6 2.7 Ad2

ALOU 2 13.51 21.21 36.43 5.6 2.7 Ad2

ALOU 3 10.89 22.16 34.73 5.56 2.5 Ad2

ALOU 4 8.64 22.9 32.88 5.7 2.9 Ad2

BRA200 1 27.46 4.26 33.97 5.4 2.5 Ad2

BRA200 2 25.91 5.64 32.67 5.33 2.26 Ad2

BRA200 3 24.97 4.59 30.81 5.45 2.5 Ad2

BRA200 4 27.22 6.05 33.7 5.65 2.5 Ad2

BRA300 1 29.91 5.32 38.11 6.1 2.9 Ad2

BRA300 2 28.69 6.08 37.11 5.85 2.55 Ad2

BRA300 3 25.98 4.8 33.2 5.53 2.4 Ad2

BRA300 4 25.9 6.02 33.37 5.52 2.5 Ad2

CRA 1 29.56 12.44 43.06 6.2 2.8 Ad2

CRA 2 33.48 12.12 47.23 6.3 2.8 Ad2

CRA 3 45.21 14.77 61.43 6.4 3 Ad2

CRA 4 27.99 11.37 40.61 6.3 2.87 Ad2

BAF 1 13.5 37.75 52.53 5 2.6 Ad2

BAF 2 13.52 45.56 61.06 5.41 2.75 Ad2

BAF 3 11.64 49.53 63.1 5.52 2.84 Ad2

BAF 4 9.77 54.73 66.18 5.7 2.9 Ad2

BM 1 9.19 43.44 54.7 5.2 2.6 Ad2

BM 2 8.25 43.43 52.89 5.2 2.5 Ad2

BM 3 9.39 39.34 49.97 5.1 2.5 Ad2

BM 4 9.36 42.98 53.49 5.2 2.6 Ad2

BUS 1 8.49 45.15 54.51 5.2 2.4 Ad2

BUS 2 7.9 51.15 60.46 5.7 2.4 Ad2

BUS 3 8.29 48.3 57.29 5.2 2.6 Ad2

BUS 4 7.86 44.98 53.9 5.2 2.3 Ad2

JJAP 1 9.79 100.82 112.28 6.02 2.85 Ad2

JJAP 2 14.6 67.86 82.81 5.87 2.88 Ad2

JJAP 3 8.21 65.82 76.8 5.71 2.69 Ad2

JJAP 4 9 51.74 61.37 5.57 2.79 Ad2

MAE 1 4.54 32.72 39.11 5.24 2.63 Ad2

MAE 2 5.97 34.49 42.41 5.12 2.54 Ad2

MAE 3 5.02 34.23 41.01 5 2.3 Ad2

MAE 4 6.05 37.64 45.23 5.16 2.56 Ad2

MOI 1 8.71 37.2 47.1 5.35 2.56 Ad2

MOI 2 7.32 54.24 63.64 5.2 2.47 Ad2

MOI 3 7.17 52.88 61.47 5.29 2.49 Ad2

MOI 4 7.01 45.15 54.16 5.23 2.46 Ad2

PER 1 15.14 28.56 44.54 5.1 2.7 Ad2

PER 2 14.39 25.77 41.34 4.8 2.5 Ad2

PER 3 11.11 30.44 42.72 5.2 2.8 Ad2

PER 4 10.51 21.15 32.58 5 2.5 Ad2

PtBas 1 10.54 35.62 47.9 6.1 2.7 Ad2

PtBas 2 8.31 46.77 57.3 6.2 2.8 Ad2

PtBas 3 9.86 70.1 82.67 6.4 3.3 Ad2

PtBas 4 9.94 52.25 64.28 6.4 3.2 Ad2

**Environnt.txt:**

asl Site habitat X.2mm pH DRXCa

100 MAL100 1 65.72 6.1 2

100 MAL100 1 65.72 6.1 2

100 MAL100 1 65.72 6.1 2

100 MAL100 1 65.72 6.1 2

200 MAL200 1 15.32 6.1 2

200 MAL200 1 15.32 6.1 2

200 MAL200 1 15.32 6.1 2

200 MAL200 1 15.32 6.1 2

300 MAL300 3 82.95 7.1 2

300 MAL300 3 82.95 7.1 2

300 MAL300 3 82.95 7.1 2

300 MAL300 3 82.95 7.1 2

400 MAL400 3 18.17 5.9 2

400 MAL400 3 18.17 5.9 2

400 MAL400 3 18.17 5.9 2

400 MAL400 3 18.17 5.9 2

500 MAL500 4 65.9 6.1 2

500 MAL500 4 65.9 6.1 2

500 MAL500 4 65.9 6.1 2

500 MAL500 4 65.9 6.1 2

600 MAL600 4 29.97 6.1 1

600 MAL600 4 29.97 6.1 1

600 MAL600 4 29.97 6.1 1

600 MAL600 4 29.97 6.1 1

700 MAL700 5 42.3 6.4 2

700 MAL700 5 42.3 6.4 2

700 MAL700 5 42.3 6.4 2

700 MAL700 5 42.3 6.4 2

800 MAL800 5 41.93 6.8 2

800 MAL800 5 41.93 6.8 2

800 MAL800 5 41.93 6.8 2

800 MAL800 5 41.93 6.8 2

200 LPN200 1 69.57 6.5 1

200 LPN200 1 69.57 6.5 1

200 LPN200 1 69.57 6.5 1

200 LPN200 1 69.57 6.5 1

400 LPN400 4 26.74 6.4 1

400 LPN400 4 26.74 6.4 1

400 LPN400 4 26.74 6.4 1

400 LPN400 4 26.74 6.4 1

600 LPN600 5 28.49 6.6 1

600 LPN600 5 28.49 6.6 1

600 LPN600 5 28.49 6.6 1

600 LPN600 5 28.49 6.6 1

700 LP700 5 66.07 6.7 2

700 LP700 5 66.07 6.7 2

700 LP700 5 66.07 6.7 2

700 LP700 5 66.07 6.7 2

600 LPS600 5 22.3 7 3

600 LPS600 5 22.3 7 3

600 LPS600 5 22.3 7 3

600 LPS600 5 22.3 7 3

550 LPS550M1 4 40.7 6.5 1

550 LPS550M1 4 40.7 6.5 1

550 LPS550M1 4 40.7 6.5 1

550 LPS550M1 4 40.7 6.5 1

550 LPS550M2 5 98.51 6.1 2

550 LPS550M2 5 98.51 6.1 2

550 LPS550M2 5 98.51 6.1 2

550 LPS550M2 5 98.51 6.1 2

400 LPS400 3 75.53 6 3

400 LPS400 3 75.53 6 3

400 LPS400 3 75.53 6 3

400 LPS400 3 75.53 6 3

300 LPS300 3 32.42 6.5 1

300 LPS300 3 32.42 6.5 1

300 LPS300 3 32.42 6.5 1

300 LPS300 3 32.42 6.5 1

300 ALOU 3 28.44 6.9 2

300 ALOU 3 28.44 6.9 2

300 ALOU 3 28.44 6.9 2

300 ALOU 3 28.44 6.9 2

200 BRA200 3 47.56 5.8 2

200 BRA200 3 47.56 5.8 2

200 BRA200 3 47.56 5.8 2

200 BRA200 3 47.56 5.8 2

300 BRA300 4 70.8 6.3 2

300 BRA300 4 70.8 6.3 2

300 BRA300 4 70.8 6.3 2

300 BRA300 4 70.8 6.3 2

500 COL 5 74.47 6.3 1

500 COL 5 74.47 6.3 1

500 COL 5 74.47 6.3 1

500 COL 5 74.47 6.3 1

800 CRA 5 40.87 6 2

800 CRA 5 40.87 6 2

800 CRA 5 40.87 6 2

800 CRA 5 40.87 6 2

100 BAF 1 46.37 7.4 3

100 BAF 1 46.37 7.4 3

100 BAF 1 46.37 7.4 3

100 BAF 1 46.37 7.4 3

20 BM 2 24.67 6.8 3

20 BM 2 24.67 6.8 3

20 BM 2 24.67 6.8 3

20 BM 2 24.67 6.8 3

20 BUS 2 13.17 5.9 1

20 BUS 2 13.17 5.9 1

20 BUS 2 13.17 5.9 1

20 BUS 2 13.17 5.9 1

20 JJAP 2 31.45 5.5 3

20 JJAP 2 31.45 5.5 3

20 JJAP 2 31.45 5.5 3

20 JJAP 2 31.45 5.5 3

80 MAE 2 38.53 8.4 3

80 MAE 2 38.53 8.4 3

80 MAE 2 38.53 8.4 3

80 MAE 2 38.53 8.4 3

50 MOI 2 53.22 7.3 3

50 MOI 2 53.22 7.3 3

50 MOI 2 53.22 7.3 3

50 MOI 2 53.22 7.3 3

260 PER 2 10 4.8 2

260 PER 2 10 4.8 2

260 PER 2 10 4.8 2

260 PER 2 10 4.8 2

50 PtBas 2 17.96 7.2 3

50 PtBas 2 17.96 7.2 3

50 PtBas 2 17.96 7.2 3

50 PtBas 2 17.96 7.2 3

100 MAL100 1 65.72 6.1 2

100 MAL100 1 65.72 6.1 2

100 MAL100 1 65.72 6.1 2

100 MAL100 1 65.72 6.1 2

200 MAL200 1 15.32 6.1 2

200 MAL200 1 15.32 6.1 2

200 MAL200 1 15.32 6.1 2

200 MAL200 1 15.32 6.1 2

300 MAL300 3 82.95 7.1 2

300 MAL300 3 82.95 7.1 2

300 MAL300 3 82.95 7.1 2

300 MAL300 3 82.95 7.1 2

400 MAL400 3 18.17 5.9 2

400 MAL400 3 18.17 5.9 2

400 MAL400 3 18.17 5.9 2

400 MAL400 3 18.17 5.9 2

500 MAL500 4 65.9 6.1 2

500 MAL500 4 65.9 6.1 2

500 MAL500 4 65.9 6.1 2

500 MAL500 4 65.9 6.1 2

600 MAL600 4 29.97 6.1 1

600 MAL600 4 29.97 6.1 1

600 MAL600 4 29.97 6.1 1

600 MAL600 4 29.97 6.1 1

700 MAL700 5 42.3 6.4 2

700 MAL700 5 42.3 6.4 2

700 MAL700 5 42.3 6.4 2

700 MAL700 5 42.3 6.4 2

800 MAL800 5 41.93 6.8 2

800 MAL800 5 41.93 6.8 2

800 MAL800 5 41.93 6.8 2

800 MAL800 5 41.93 6.8 2

700 LP700 5 66.07 6.7 2

700 LP700 5 66.07 6.7 2

700 LP700 5 66.07 6.7 2

700 LP700 5 66.07 6.7 2

550 LPS550M1 4 40.7 6.5 1

550 LPS550M1 4 40.7 6.5 1

550 LPS550M1 4 40.7 6.5 1

550 LPS550M1 4 40.7 6.5 1

550 LPS550M2 5 98.51 6.1 2

550 LPS550M2 5 98.51 6.1 2

550 LPS550M2 5 98.51 6.1 2

550 LPS550M2 5 98.51 6.1 2

400 LPS400 3 75.53 6 3

400 LPS400 3 75.53 6 3

400 LPS400 3 75.53 6 3

400 LPS400 3 75.53 6 3

300 LPS300 3 32.42 6.5 1

300 LPS300 3 32.42 6.5 1

300 LPS300 3 32.42 6.5 1

300 LPS300 3 32.42 6.5 1

300 ALOU 3 28.44 6.9 2

300 ALOU 3 28.44 6.9 2

300 ALOU 3 28.44 6.9 2

300 ALOU 3 28.44 6.9 2

200 BRA200 3 47.56 5.8 2

200 BRA200 3 47.56 5.8 2

200 BRA200 3 47.56 5.8 2

200 BRA200 3 47.56 5.8 2

300 BRA300 4 70.8 6.3 2

300 BRA300 4 70.8 6.3 2

300 BRA300 4 70.8 6.3 2

300 BRA300 4 70.8 6.3 2

800 CRA 5 40.87 6 2

800 CRA 5 40.87 6 2

800 CRA 5 40.87 6 2

800 CRA 5 40.87 6 2

100 BAF 1 46.37 7.4 3

100 BAF 1 46.37 7.4 3

100 BAF 1 46.37 7.4 3

100 BAF 1 46.37 7.4 3

20 BM 2 24.67 6.8 3

20 BM 2 24.67 6.8 3

20 BM 2 24.67 6.8 3

20 BM 2 24.67 6.8 3

20 BUS 2 13.17 5.9 1

20 BUS 2 13.17 5.9 1

20 BUS 2 13.17 5.9 1

20 BUS 2 13.17 5.9 1

20 JJAP 2 31.45 5.5 3

20 JJAP 2 31.45 5.5 3

20 JJAP 2 31.45 5.5 3

20 JJAP 2 31.45 5.5 3

80 MAE 2 38.53 8.4 3

80 MAE 2 38.53 8.4 3

80 MAE 2 38.53 8.4 3

80 MAE 2 38.53 8.4 3

50 MOI 2 53.22 7.3 3

50 MOI 2 53.22 7.3 3

50 MOI 2 53.22 7.3 3

50 MOI 2 53.22 7.3 3

260 PER 2 10 4.8 2

260 PER 2 10 4.8 2

260 PER 2 10 4.8 2

260 PER 2 10 4.8 2

50 PtBas 2 17.96 7.2 3

50 PtBas 2 17.96 7.2 3

50 PtBas 2 17.96 7.2 3

50 PtBas 2 17.96 7.2 3
