# Supplementary material for: Soil Calcium Availability Influences Shell Ecophenotype Formation in the Sub-Antarctic Land Snail, Notodiscus hookeri
Source: PLoS One. 2013 Dec 20;8(12):e84527. doi: 10.1371/journal.pone.0084527 (PMC3869943; doi:10.1371/journal.pone.0084527)
Supplement: Text S7 — Scripts used for the RDA and for the MANOVA after RDA. (DOCX) [file pone.0084527.s007.docx]

**Text. S7: The scripts used for the RDA and for the MANOVA after RDA**

**1) SCRIPTS USED FOR RDA WITH R**

#==============================================================

ad12=read.table("ad12.txt",header=T)

ad12$indiv=factor(ad12$indiv)

summary(ad12)

#==============================================================

# from Environnt.txt 220 lines

#==============================================================

envir=read.table("envir.txt",header=T)

summary(envir)

envir$habitat=as.factor(envir$habitat)

envir$DRXCa=as.factor(envir$DRXCa)

summary(envir)

#==============================================================

# Construction of a matrix X for the Rda

#==============================================================

attach(envir)

type=ad12$type

X=data.frame(asl,X.2mm,pH,habitat,DRXCa,type,row.names=envir$Sites)

detach(envir)

summary(X)

#==============================================================

# CALCULATION. 1 - RDA (acp-vi)

#==============================================================

library(ade4)

#========================================================

# PHASE 1 : ACP of the Table Y

#========================================================

Y=ad12[,3:7]

ad12.dudi=dudi.pca(Y)

par(mfrow=c(2,2))

s.class(ad12.dudi$li,fac=envir$Site,clabel=0.5,cpoint=2,pch=20,col=as.numeric(envir$Site))

s.arrow(ad12.dudi$c1, lab=names(Y))

s.class(ad12.dudi$li,fac=envir$Site:ad12$type,label=NULL,cpoint=2,pch=20,col=as.numeric(envir$Site))

s.corcircle(ad1.dudi$co,lab=names(Y))

par(mfrow=c(1,1))

#========================================================

# PHASE 2 : ACP-VI or RDA of Y on X

#========================================================

ad12.pcaiv=pcaiv(ad12.dudi,X)

summary(ad12.pcaiv)

plot(ad12.pcaiv)

drda=dist.dudi(ad12.pcaiv)

cahrda=hclust(drda,"ward")

nn=length(cahrda$height)

barplot(cahrda$height[nn:180]);title("distances between the nodes")

#cahrda=pvclust(ad1.dudi$li,method.hclust="ward",method.dist="euclidian",r=0.1,nboot=500)

plot(cahrda);windows()

windows()

#==============================================================

# CALCULATION. 2 – MONTE-CARLO TEST

#==============================================================

rand2<-randtest(ad1.pcaiv,nrepet=1000)

plot(rand2,main="Monte Carlo Test on RDA")

rand2

#==============================================================

# CALCULATION. 3 – Adding the groups

#==============================================================

Group=as.factor(cutree(cahrda, h = 20))

levels(Group)=c("OS1","MS1","OS2","MS2")

u=s.class(dfxy=ad12.dudi$li,fac=Group,col=c(1,2,3,4),add.plot=F)

u=s.class(dfxy=ad12.dudi$li,fac=type,col=c(1,2,3,4),add.plot=F)

#==============================================================

# CALCULATION. 4 – Position of the gravity centers for each snail

#==============================================================

s.class(dfxy=ad12.dudi$li,fac=envir$Site:type,cellipse=0,clabel=0.5,cstar=0,cpoint=0,col=1:60,add.plot=F)

coin1=locator(1)

coin2=locator(1)

xmin=coin1$x;xmax=coin2$x

ymin=coin1$y;ymax=coin2$y

xmin

s.class(dfxy=ad12.dudi$li,fac=envir$Site:type,cellipse=0,clabel=0.5,cstar=0,cpoint=0,col=as.numeric(Site),xlim=c(xmin,xmax),ylim=c(ymin,ymax),add.plot=F)

s.class(dfxy=ad12.dudi$li,fac=Group,clabel=0.5,cstar=0,cpoint=0,col=1:4,xlim=c(xmin,xmax),ylim=c(ymin,ymax),add.plot=T)

#==============================================================

# CALCULATION. 5 – PERCENTAGE OF INERTIA EXPLAINED BY THE RDA

#==============================================================

trace1=sum(ad12.dudi$eig);trace1 trace2=sum(ad12.pcaiv$eig);trace2 ratio=trace2/trace1;ratio pourcent=ratio*100;cat("pourcentage :",pourcent,"\n")

**2) SCRIPTS USED FOR MANOVA WITH R**

#==============================================================

# CALCULATION. 1 - MANOVA

#==============================================================

attach(X)

summary(X)

summary(Y)

m1=manova(as.matrix(Y)~X.2mm+pH+Group+Site:habitat+habitat+DRXCa)

sm1=summary(m1)

m2=manova(as.matrix(Y)~X.2mm+pH+Site:habitat+habitat+habitat*Group+DRXCa)

summary(m2)

m3=manova(as.matrix(Y)~X.2mm+pH+Group+Site:habitat+habitat*DRXCa*Group)

summary(m3)

m4=manova(as.matrix(Y)~X.2mm+pH+Group+Site:habitat+habitat:DRXCa+Group:DRXCa)

summary(m4)

m31=manova(as.matrix(Y)~X.2mm+pH+Group+Site:habitat+habitat*DRXCa*Group+asl)

summary(m31)

AIC(m1,m2,m3,m31,m4)

lm1=lm(Y$OL~X.2mm+X1mm+X0.5mm+X0.2mm+X0.1mm+pH+ad1.Group+Site:habitat+habitat+DRXCa)

anova(lm1)

lm2=lm(Y$OL~X.2mm+X0.5mm+X0.2mm+X0.1mm+pH+ad1.Group+Site:habitat+habitat+DRXCa)

anova(lm2)

lm3=lm(Y$OL~X.2mm+X0.5mm+X0.2mm+pH+ad1.Group+Site:habitat+habitat+DRXCa)

anova(lm3)

lm4=lm(Y$OL~X.2mm+X0.2mm+pH+ad1.Group+Site:habitat+habitat+DRXCa)

anova(lm4)

AIC(lm1,lm2,lm3,lm4)

#==============================================================

# CALCULATION. 2 –TEST OF PILLAI WITH THE SITE AS A RANDOM FACTOR

#==============================================================

suptest = function (m,randfac)

{

smm=summary(m)

sm=data.frame(summary(m)$stats)

names=row.names(sm)

randfacnumber=which(names==randfac)

if(class(m)[1]!="manova") stop("Pas MANOVA !") else cat("MANOVA on the random factor : ",as.character(randfac),"\n")

k=dim(sm)[1]-2

nlig=k+2

u=NULL

list(randfacnumber,k)

V=F=dF1=dF2=pvalue=rep(0,k)

norder=(1:nlig)[names != randfac & names != "Residuals"]

nnames=names[names != randfac & names != "Residuals"]

W=smm$SS[[randfacnumber]]

nu=sm$Df[nlig]

sgn=rep("",k)

for(i in 1:k)

{

#************************** Calculation of the Pillai test ***************

B=smm$SS[[norder[i]]]

V[i]=sum(diag(B %*% solve(B+W)))

#************************** Calculation of F Value approximation ***************

p=dim(B)[1]

q=sm$Df[norder[i]]

m=(abs(p-q)-1)/2

n=(nu-p-1)/2

s=min(p,q)

F[i]=(2*n+s+1)*V[i]/(2*m+s+1)/(s-V[i])

dF1[i]=s*(2*m+s+1)

dF2[i]=s*(2*2+s+1)

pvalue[i]=1-pf(F[i],dF1[i],dF2[i])

unpourmille=pvalue[i]<=0.001

unpourcent=pvalue[i]<=0.01

cinqpourcent=pvalue[i]<=0.05

dixpourcent=pvalue[i]<=0.1

sgn[i]=if(unpourmille) "***" else if(unpourcent) "**" else if(cinqpourcent) "*" else if(dixpourcent) "." else ""

}

df=data.frame(nnames,norder,V,F,dF1,dF2,pvalue,sgn)

names(df)=c("Effect","number","Pillai","F","df1","df2","pvalue","")

df

}

suptest(m4,"Site:habitat")
