# Supplementary material for: Soil Calcium Availability Influences Shell Ecophenotype Formation in the Sub-Antarctic Land Snail, Notodiscus hookeri
Source: PLoS One. 2013 Dec 20;8(12):e84527. doi: 10.1371/journal.pone.0084527 (PMC3869943; doi:10.1371/journal.pone.0084527)
Supplement: Text S8 — Data set (Corr Size_Thick.txt) used to carry out ANCOVA. (DOCX) [file pone.0084527.s008.docx]

**Text. S8: The data set (Corr Size_Thick.txt) used to carry out ANCOVA**

SITE TYPE Size Aper DRXCa

MAL100 ad1 4.6 2.4 NoclayNoCa

MAL100 ad1 4.5 2.3 NoclayNoCa

MAL100 ad1 4.5 2.2 NoclayNoCa

MAL100 ad1 4.1 2.3 NoclayNoCa

MAL200 ad1 4.2 2 NoclayNoCa

MAL200 ad1 4.2 2.1 NoclayNoCa

MAL200 ad1 4.1 2.1 NoclayNoCa

MAL200 ad1 4 2.1 NoclayNoCa

MAL300 ad1 4.3 2.4 NoclayNoCa

MAL300 ad1 4.6 2.5 NoclayNoCa

MAL300 ad1 4.7 2.5 NoclayNoCa

MAL300 ad1 4.6 2.5 NoclayNoCa

MAL400 ad1 4.71 2.43 NoclayNoCa

MAL400 ad1 4.64 2.48 NoclayNoCa

MAL400 ad1 4.74 2.46 NoclayNoCa

MAL400 ad1 4.2 2.3 NoclayNoCa

MAL500 ad1 4.3 2.3 NoclayNoCa

MAL500 ad1 4.34 2.4 NoclayNoCa

MAL500 ad1 4.35 2.2 NoclayNoCa

MAL500 ad1 4.25 2.15 NoclayNoCa

MAL600 ad1 4.15 1.9 Clay

MAL600 ad1 4.36 2.55 Clay

MAL600 ad1 4.09 2.12 Clay

MAL600 ad1 4.43 2.31 Clay

MAL700 ad1 4 2.2 NoclayNoCa

MAL700 ad1 4.55 2.43 NoclayNoCa

MAL700 ad1 4.8 2.5 NoclayNoCa

MAL700 ad1 4.38 2.41 NoclayNoCa

MAL800 ad1 4.53 2.6 NoclayNoCa

MAL800 ad1 4.7 2.7 NoclayNoCa

MAL800 ad1 4.47 2.49 NoclayNoCa

MAL800 ad1 4.3 2.43 NoclayNoCa

LPN200 ad1 4.1 2 Clay

LPN200 ad1 4.6 2.4 Clay

LPN200 ad1 4 2 Clay

LPN200 ad1 4.1 2.1 Clay

LPN400 ad1 4 1.7 Clay

LPN400 ad1 4.2 2 Clay

LPN400 ad1 4.7 2.5 Clay

LPN400 ad1 4 1.9 Clay

LPN600 ad1 4.3 2 Clay

LPN600 ad1 4.8 2.5 Clay

LPN600 ad1 4 2 Clay

LPN600 ad1 4.61 2.1 Clay

LP700 ad1 4.3 2.4 NoclayNoCa

LP700 ad1 4.2 2.1 NoclayNoCa

LP700 ad1 4 2 NoclayNoCa

LP700 ad1 4.1 2.3 NoclayNoCa

LPS600 ad1 4 1.8 Ca

LPS600 ad1 4 1.9 Ca

LPS600 ad1 4.1 2.1 Ca

LPS600 ad1 4.2 2 Ca

LPS550M1 ad1 4.5 2.5 Clay

LPS550M1 ad1 4.3 2.2 Clay

LPS550M1 ad1 4.1 1.9 Clay

LPS550M1 ad1 4 1.9 Clay

LPS550M2 ad1 4.6 2.3 NoclayNoCa

LPS550M2 ad1 4.7 2.3 NoclayNoCa

LPS550M2 ad1 4.6 2.4 NoclayNoCa

LPS550M2 ad1 4.2 2.1 NoclayNoCa

LPS400 ad1 4.1 1.9 Ca

LPS400 ad1 4.1 1.9 Ca

LPS400 ad1 4.2 2.2 Ca

LPS400 ad1 4.6 2.1 Ca

LPS300 ad1 4.1 1.8 Clay

LPS300 ad1 4 1.9 Clay

LPS300 ad1 4.2 2.1 Clay

LPS300 ad1 4.1 1.8 Clay

ALOU ad1 4.6 2.4 NoclayNoCa

ALOU ad1 4.3 2.4 NoclayNoCa

ALOU ad1 4.2 2 NoclayNoCa

ALOU ad1 4.4 2.2 NoclayNoCa

BRA200 ad1 4.61 2.62 NoclayNoCa

BRA200 ad1 4.6 2.5 NoclayNoCa

BRA200 ad1 4.31 2.32 NoclayNoCa

BRA200 ad1 4.05 2.1 NoclayNoCa

BRA300 ad1 4.7 2.1 NoclayNoCa

BRA300 ad1 4.54 2.34 NoclayNoCa

BRA300 ad1 4.5 2.25 NoclayNoCa

BRA300 ad1 4.3 2.2 NoclayNoCa

COL ad1 4.3 2 Clay

COL ad1 4.6 2.1 Clay

COL ad1 4.2 1.9 Clay

COL ad1 4.3 2.1 Clay

CRA ad1 4.6 2.5 NoclayNoCa

CRA ad1 4.4 2.4 NoclayNoCa

CRA ad1 4.5 2.43 NoclayNoCa

CRA ad1 4.5 2.4 NoclayNoCa

BAF ad1 4.1 2 Ca

BAF ad1 4.1 2.12 Ca

BAF ad1 4.13 2.25 Ca

BAF ad1 4.08 1.91 Ca

BM ad1 4 2.1 Ca

BM ad1 4.3 2.3 Ca

BM ad1 4.3 2.4 Ca

BM ad1 4 2.1 Ca

BUS ad1 4.4 2.4 Clay

BUS ad1 4.1 2 Clay

BUS ad1 4.2 2.1 Clay

BUS ad1 4.4 2.2 Clay

JJAP ad1 4.19 2.27 Ca

JJAP ad1 4.26 2.2 Ca

JJAP ad1 4.13 2.39 Ca

JJAP ad1 4.39 2.18 Ca

MAE ad1 4.2 2.12 Ca

MAE ad1 4.29 2.16 Ca

MAE ad1 4.42 2.21 Ca

MAE ad1 4.1 2.3 Ca

MOI ad1 3.83 2.02 Ca

MOI ad1 4.3 2.1 Ca

MOI ad1 4.25 2.17 Ca

MOI ad1 4.07 2.01 Ca

PER ad1 4.1 2.3 NoclayNoCa

PER ad1 3.9 2 NoclayNoCa

PER ad1 4 2.2 NoclayNoCa

PER ad1 4 2 NoclayNoCa

PtBas ad1 4.4 2.3 Ca

PtBas ad1 4.4 2.3 Ca

PtBas ad1 4.6 2.4 Ca

PtBas ad1 4.3 2.2 Ca

MAL100 ad2 6.4 3 NoclayNoCa

MAL100 ad2 5.5 2.6 NoclayNoCa

MAL100 ad2 5.9 2.8 NoclayNoCa

MAL100 ad2 5.5 2.7 NoclayNoCa

MAL200 ad2 6 2.9 NoclayNoCa

MAL200 ad2 5.7 2.7 NoclayNoCa

MAL200 ad2 5.7 2.7 NoclayNoCa

MAL200 ad2 5.2 2.8 NoclayNoCa

MAL300 ad2 5.9 2.7 NoclayNoCa

MAL300 ad2 6.1 2.8 NoclayNoCa

MAL300 ad2 6 2.8 NoclayNoCa

MAL300 ad2 6 2.8 NoclayNoCa

MAL400 ad2 6.77 3.2 NoclayNoCa

MAL400 ad2 6.3 3.1 NoclayNoCa

MAL400 ad2 6.18 3.01 NoclayNoCa

MAL400 ad2 6.9 3.4 NoclayNoCa

MAL500 ad2 6.2 2.8 NoclayNoCa

MAL500 ad2 5.83 2.8 NoclayNoCa

MAL500 ad2 5.7 2.7 NoclayNoCa

MAL500 ad2 5.8 2.6 NoclayNoCa

MAL600 ad2 6.54 3.42 Clay

MAL600 ad2 6.28 3 Clay

MAL600 ad2 6.6 3.2 Clay

MAL600 ad2 6.1 2.72 Clay

MAL700 ad2 6 3 NoclayNoCa

MAL700 ad2 6.08 3.15 NoclayNoCa

MAL700 ad2 6.19 3.07 NoclayNoCa

MAL700 ad2 5.95 2.79 NoclayNoCa

MAL800 ad2 7.21 3.19 NoclayNoCa

MAL800 ad2 6.67 2.96 NoclayNoCa

MAL800 ad2 6.47 3 NoclayNoCa

MAL800 ad2 6.57 3.07 NoclayNoCa

LP700 ad2 7 3.2 NoclayNoCa

LP700 ad2 7 3.3 NoclayNoCa

LP700 ad2 7 3.3 NoclayNoCa

LP700 ad2 6.8 3 NoclayNoCa

LPS550M1 ad2 5.7 2.8 Clay

LPS550M1 ad2 5.4 2.6 Clay

LPS550M1 ad2 5.6 2.5 Clay

LPS550M1 ad2 5.4 2.6 Clay

LPS550M2 ad2 7.1 2.8 NoclayNoCa

LPS550M2 ad2 6.4 2.7 NoclayNoCa

LPS550M2 ad2 6.1 2.8 NoclayNoCa

LPS550M2 ad2 6.53 2.7 NoclayNoCa

LPS400 ad2 5.6 2.7 Ca

LPS400 ad2 5.2 2.4 Ca

LPS400 ad2 5.88 2.9 Ca

LPS400 ad2 5.3 2.6 Ca

LPS300 ad2 5.3 2.5 Clay

LPS300 ad2 5.3 2.5 Clay

LPS300 ad2 5.3 2.2 Clay

LPS300 ad2 5.2 2.4 Clay

ALOU ad2 5.6 2.7 NoclayNoCa

ALOU ad2 5.6 2.7 NoclayNoCa

ALOU ad2 5.56 2.5 NoclayNoCa

ALOU ad2 5.7 2.9 NoclayNoCa

BRA200 ad2 5.4 2.5 NoclayNoCa

BRA200 ad2 5.33 2.26 NoclayNoCa

BRA200 ad2 5.45 2.5 NoclayNoCa

BRA200 ad2 5.65 2.5 NoclayNoCa

BRA300 ad2 6.1 2.9 NoclayNoCa

BRA300 ad2 5.85 2.55 NoclayNoCa

BRA300 ad2 5.53 2.4 NoclayNoCa

BRA300 ad2 5.52 2.5 NoclayNoCa

CRA ad2 6.2 2.8 NoclayNoCa

CRA ad2 6.3 2.8 NoclayNoCa

CRA ad2 6.4 3 NoclayNoCa

CRA ad2 6.3 2.87 NoclayNoCa

BAF ad2 5 2.6 Ca

BAF ad2 5.41 2.75 Ca

BAF ad2 5.52 2.84 Ca

BAF ad2 5.7 2.9 Ca

BM ad2 5.2 2.6 Ca

BM ad2 5.2 2.5 Ca

BM ad2 5.1 2.5 Ca

BM ad2 5.2 2.6 Ca

BUS ad2 5.2 2.4 Clay

BUS ad2 5.7 2.4 Clay

BUS ad2 5.2 2.6 Clay

BUS ad2 5.2 2.3 Clay

JJAP ad2 6.02 2.85 Ca

JJAP ad2 5.87 2.88 Ca

JJAP ad2 5.71 2.69 Ca

JJAP ad2 5.57 2.79 Ca

MAE ad2 5.24 2.63 Ca

MAE ad2 5.12 2.54 Ca

MAE ad2 5 2.3 Ca

MAE ad2 5.16 2.56 Ca

MOI ad2 5.35 2.56 Ca

MOI ad2 5.2 2.47 Ca

MOI ad2 5.29 2.49 Ca

MOI ad2 5.23 2.46 Ca

PER ad2 5.1 2.7 NoclayNoCa

PER ad2 4.8 2.5 NoclayNoCa

PER ad2 5.2 2.8 NoclayNoCa

PER ad2 5 2.5 NoclayNoCa

PtBas ad2 6.1 2.7 Ca

PtBas ad2 6.2 2.8 Ca

PtBas ad2 6.4 3.3 Ca

PtBas ad2 6.4 3.2 Ca
