# Supplementary material for: Soil Calcium Availability Influences Shell Ecophenotype Formation in the Sub-Antarctic Land Snail, Notodiscus hookeri
Source: PLoS One. 2013 Dec 20;8(12):e84527. doi: 10.1371/journal.pone.0084527 (PMC3869943; doi:10.1371/journal.pone.0084527)
Supplement: Text S9 — Summary of ANCOVA performed on shell size and shell thickness according to soil mineralogy. (DOCX) [file pone.0084527.s009.docx]

**Text. S9: Summary of the ANCOVA performed on shell size and shell thickness according to soil mineralogy.**

Models

> m1=lm(Thick~DRXCa*Size)

> m2=lm(Thick~DRXCa:Size)

- m3=lm(Thick~DRXCa+DRXCa:Size)

Choice of the best model :

- > AIC(m1,m2,m3)
- df AIC
- m1 7 1541.922
- m2 5 1568.085
- m3 7 1541.922

> summary(m3)

Call:

lm(formula = Thick ~ DRXCa + DRXCa:Size)

Residuals:

Min 1Q Median 3Q Max

-19.638 -4.412 0.003 3.802 47.251

Coefficients:

Estimate Std. Error t value Pr(>|t|)

(Intercept) -25.7691 6.8932 -3.738 0.000238 ***

DRXCaClay 44.3278 10.1964 4.347 2.13e-05 ***

DRXCaNoclay 43.7519 8.1060 5.397 1.79e-07 ***

DRXCaCa:Size 15.0828 1.4173 10.642 < 2e-16 ***

DRXCaClay:Size 3.8807 1.5754 2.463 0.014558 *

DRXCaNoclay:Size 3.5514 0.8071 4.400 1.71e-05 ***

---

Signif. codes: 0 ‘***’ 0.001 ‘**’ 0.01 ‘*’ 0.05 ‘.’ 0.1 ‘ ’ 1

Residual standard error: 7.905 on 214 degrees of freedom

Multiple R-squared: 0.4971, Adjusted R-squared: 0.4853

F-statistic: 42.3 on 5 and 214 DF, p-value: < 2.2e-16

Correlations

# Ca^+^ : y= 15.0828 x - 25.7691

# Clay : y=3.8807 x + 44.32 - 25.7691 (intercept à 18.5509)

# Noclay (Ca^-^): y= 3.5514 x + 43.75 - 25.7691 (intercept à 17.9809)

> anova(m3)

Analysis of Variance Table

Response: Thick

Df Sum Sq Mean Sq F value Pr(>F)

DRXCa 2 4549.0 2274.50 36.403 2.469e-14 ***

DRXCa:Size 3 8665.4 2888.46 46.229 < 2.2e-16 ***

Residuals 214 13371.1 62.48
