# Supplementary figures and images for: Soil Calcium Availability Influences Shell Ecophenotype Formation in the Sub-Antarctic Land Snail, Notodiscus hookeri
Source: PLoS One. 2013 Dec 20;8(12):e84527. doi: 10.1371/journal.pone.0084527 (PMC3869943; doi:10.1371/journal.pone.0084527)

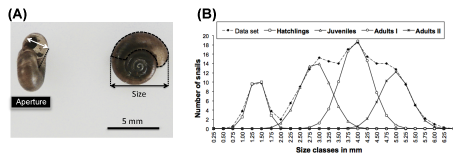

Supplement: Figure S1 — Features of shell measurements performed on the snail Notodiscus hookeri. Size = the widest diagonal of the shell; Aperture = the widest diagonal of the shell aperture. The dotted part is the last whorl used to study shell micro-scale structure according to age (A) and cohorts of the snail Notodiscus hookeri during the summer recruitment period (January–April, n = 210), from a population collected in 2006 at Base Alfred Faure on Possession Island (B). Size classes are defined by shell size measurements. Four cohorts were delineated, based on shell sizes (mean in mm ± Sd) and relative contribution (in %) to the snail population: Hatchlings: 1.38 ± 0.21 (12 %); Juveniles: 2.90 ± 0.40 (28 %); Adults 1 (young): 3.96 ± 0.40 (37 %) and Adults 2 (older): 4.98 ± 0.38 (23 %). (TIF) [file pone.0084527.s010.tif]

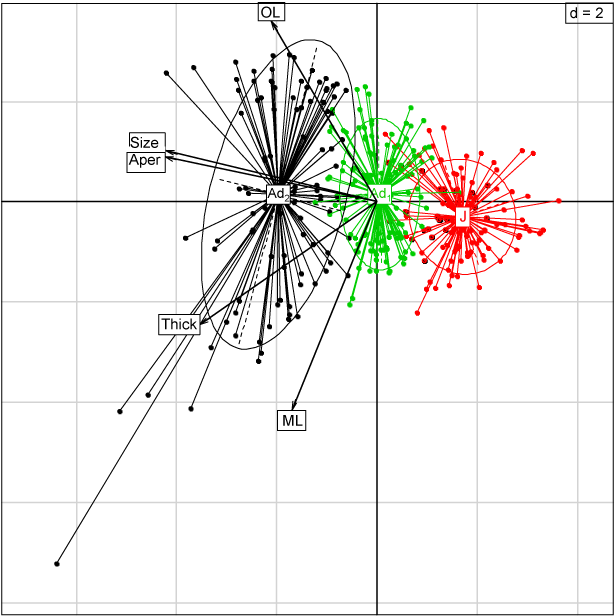

Supplement: Figure S2 — Principal Component Analysis (PCA) of shell parameters in both juveniles and adults of Notodiscus hookeri. Four snails were sampled per site (n = 30 sites), but no juveniles were collected at the sites LP700 and CRA and no adult-2 snails were collected at the sites LPN 200, LPN 400, LPN 600, LPS600 and COL. Therefore the number of snails analysed was n = 332. The two first components axes of the PCA are presented. The shell parameters are symbolized by arrows and are superimposed to the individuals J (juveniles), Ad1 (adult-1) and Ad2 (adult-2). The 95% confidence ellipses on the graph illustrate the three cohorts where each individual is represented by a dot. In support to our argumentation in the main text, we can see that Juveniles are altogether in one group. The ecophenotypes make discrete clusters in adult-1 snails and are clearly delineated in adult-2 snails. Therefore, the differentiation in two ecophenotypes appears progressively with age. For abbreviations, see the Figures 1 and 4. (TIF) [file pone.0084527.s011.tif]

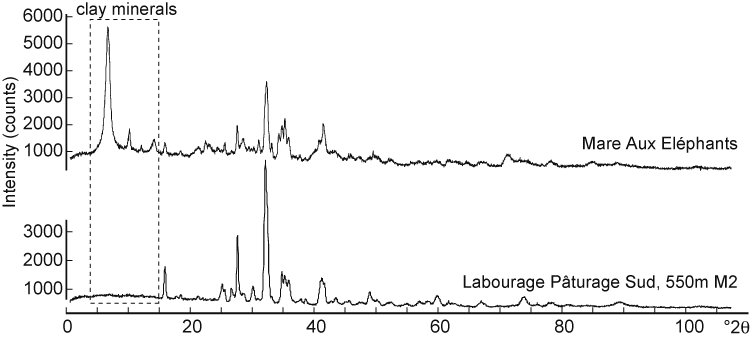

Supplement: Figure S3 — X ray diffraction powder patterns of the < 0.1 mm fractions in soils. The samples are from "Mare Aux Éléphants" and "Labourage Pâturage Sud, 550 m M2". The rectangular dotted line indicates the region of interest for the investigation of clay minerals. Other peaks (> 15 °2θ) are attributed to feldspars, magnetite and mica. (TIF) [file pone.0084527.s012.tif]

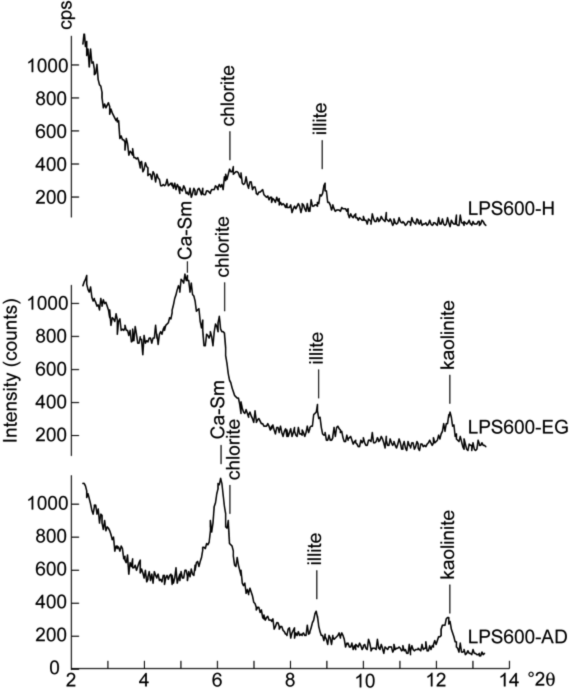

Supplement: Figure S4 — X ray diffraction oriented patterns of the <2 µm fractions in soils. The sample from "LabouragePâturageSud, 600 m Sud" is given as an example. -AD: air dried conditions, -EG: saturated with ethylene glycol, -H: heated to 550 °C. Structural formulae are:. for Ca-rich smectite (Ca-Sm),. for Chlorite,. Si4(Al2-xR2+x)Ca x/2 O 10(OH)2 nH 2 O for CA-rich smectite(Ca-Sm), (Si4-xAlx)(R2+3)(R3+xR2+3-x)O 10(OH)8 for Chlorite, (Si4-xAlx)Al2KxO10(OH)2 for Illite and Si 2 Al 2 O 5(OH)4 for Kaolinite. R2+ = Mg2+, Fe2+, Mn2+; R3+ = Al3+, Fe3. (TIF) [file pone.0084527.s013.tif]

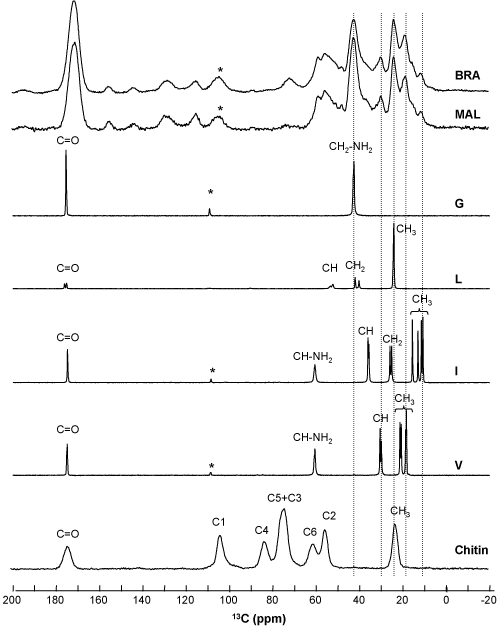

Supplement: Figure S5 — {1H}13C CPMAS spectrum of the organic layer of Notodiscus hookeri shells. Shells originated from Malpassée (MAL) were compared with those from Branca (BRA) and put in relation to the spectra of ß-chitin powder and of the most abundant L-amino acids found in this layer (G = glycine, L = leucine, I = isoleucine and V = valine). For other explanations, see Figure 3. (TIF) [file pone.0084527.s014.tif]
