# Supplementary material for: Soil Calcium Availability Influences Shell Ecophenotype Formation in the Sub-Antarctic Land Snail, Notodiscus hookeri
Source: PLoS One. 2013 Dec 20;8(12):e84527. doi: 10.1371/journal.pone.0084527 (PMC3869943; doi:10.1371/journal.pone.0084527)
Supplement: Table S1 — (DOCX [file pone.0084527.s015.docx]

Table S1. Partial peptide sequences of the organic insoluble matrix in *Notodiscus hookeri* shell. Sequences were deduced from MS/MS spectra.
